# Neuronal fate specification by the Dbx1 transcription factor is linked to the evolutionary acquisition of a novel functional domain

**DOI:** 10.1186/s13227-016-0055-5

**Published:** 2016-08-12

**Authors:** Sonia Karaz, Maximilien Courgeon, Hélène Lepetit, Eugenia Bruno, Raimondo Pannone, Andrea Tarallo, France Thouzé, Pierre Kerner, Michel Vervoort, Frédéric Causeret, Alessandra Pierani, Giuseppe D’Onofrio

**Affiliations:** 1 Institut Jacques Monod, CNRS UMR 7592, Université Paris Diderot, Sorbonne Paris Cité, 75205 Paris Cedex, France; 2 Dept. BEOM, Stazione Zoologica A. Dohrn, Villa Comunale, 80121 Naples, Italy

**Keywords:** Nervous system, Spinal cord, Neuronal identity, Transcription factor, Protein domains

## Abstract

**Background:**

Dbx1 is a homeodomain transcription factor involved in neuronal fate specification belonging to a widely conserved family among bilaterians. In mammals, Dbx1 was proposed to act as a transcriptional repressor by interacting with the Groucho corepressors to allow the specification of neurons involved in essential biological functions such as locomotion or breathing.

**Results:**

Sequence alignments of Dbx1 proteins from different species allowed us to identify two conserved domains related to the Groucho-dependent Engrailed repressor domain (RD), as well as a newly described domain composed of clusterized acidic residues at the C-terminus (Cter) which is present in tetrapods but also several invertebrates. Using a heterologous luciferase assay, we showed that the two putative repressor domains behave as such in a Groucho-dependent manner, whereas the Cter does not bear any intrinsic transcriptional activity. Consistently with in vitro data, we found that both RDs are involved in cell fate specification using in vivo electroporation experiments in the chick spinal cord. Surprisingly, we show that the Cter domain is required for Dbx1 function in vivo, acting as a modulator of its repressive activity and/or imparting specificity.

**Conclusion:**

Our results strongly suggest that the presence of a Cter domain among tetrapods is essential for Dbx1 to regulate neuronal diversity and, in turn, nervous system complexity.

**Electronic supplementary material:**

The online version of this article (doi:10.1186/s13227-016-0055-5) contains supplementary material, which is available to authorized users.

## Background

In the eumetazoan group, development of the nervous system relies on the progressive diversification of neuronal cell types and the establishment of appropriate connections between them. The architecture of the nervous system is therefore shaped by evolutionary constraints. For instance, transition from aquatic to terrestrial life was accompanied by an increase in cell diversity and circuit complexity specifically allowing breathing and locomotion. Neuronal cell identity and connectivity are regulated by complex gene regulatory networks that typically involve homeodomain (HD) transcription factors (TFs). The developmental expression and interaction of HD TFs with one another are evolutionarily conserved and tightly regulated in order to ensure the spatial and temporal coordination of target gene expression.

Members of the Dbx family of HD TFs have been identified in a range of bilaterian species. *Dbx* genes are expressed in the developing nervous system [1–7] and were shown to be involved in neuronal cell fate determination in *Drosophila* [3], zebrafish [8, 9], *Xenopus* [2, 5] and mice [10]. In the murine spinal cord, Dbx1 coordinates the differentiation of neurons essential for the alternation of left and right limbs, thus allowing locomotion [11, 12]. Mouse Dbx1 is also required to control the identity and function of neurons which generate synchronous breathing rhythms in the rhombencephalon [13, 14]. More recently, Dbx1 has been shown to play a critical role in the specification of hypothalamic neurons governing innate stress circuits which include predator avoidance and feeding [15]. Dbx1 therefore controls the formation of neural networks governing physiological functions which were fundamental during mammalian evolution.

Most HD TFs controlling cell fate in the vertebrate spinal cord (including Dbx1 and Dbx2) are thought to act as transcriptional repressors via an Engrailed homology-1 (eh1) domain which recruits the co-repressor Groucho [16]. This has led to the “derepression” model: Cell identity in the spinal cord is assigned by the derepression of effector genes [17]. It has thus been inferred that only two kinds of domains, namely DNA-binding HD and eh1-like repressor domains, mediate Dbx1/2 functions.

Protein domains are defined regions of a polypeptide structure that often carry specific functions. Hence, the “domain architecture” of a protein represents a primary level to understand its function(s) [18]. The vast majority of prokaryotic and eukaryotic gene products carry two or more domains [19]. Interestingly, it was reported that the complexity of an organism is more related to the combinatorial organization of protein domains created by domain shuffling, i.e., domain architecture complexity, than with the gene number harbored in the genome [20]. Thus, protein evolution could be better understood analyzing the evolution of domain architecture, since a domain sequence by means of mutations, insertions or deletions could become a new domain with close or even different function from the original one [21]. However, the identification of protein domains based on sequence alone remains a challenging task [22].

Here, we analyzed a multiple alignment of Dbx protein family members found in a representative range of bilaterians. In addition to previously suggested putative repressor domains (RDs), we identified a novel domain enriched in acidic residues at the C-terminus (Cter domain) highly conserved among tetrapods, but also found in several lineages among bilaterians, suggesting it yields an evolutionary conserved crucial function. We implemented in vitro luciferase reporter assays to assess the intrinsic transcriptional activity of Dbx1 domains and further tested their contribution to the in vivo function of the protein using chick *in ovo* electroporation. These experiments allowed us to demonstrate that the newly identified Cter domain is critical to regulate fate specification properties of Dbx1. We propose that the strong conservation of the Cter domain of Dbx1 among tetrapods reveals its contribution to the regulation of neuronal diversity and nervous system complexification during evolution.

## Methods

### Sequences, alignment and protein structure

The following sequences of Dbx proteins family were retrieved from NCBI [23], Aniseed [24] or Uniprot [25], and European Nucleotide Archive [26]. The species names and the accession numbers of Dbx proteins are the following: human (*Homo sapiens* Dbx1: NP_001025036.2; Dbx2: NP_001004329.2), macaque (*Macaca fascicularis* Dbx1: XP_005578455.1; Dbx2: XP_005570688.1), mouse (*Mus musculus* Dbx1: NP_001005232.1; Dbx2: NP_997416.2), opossum (*Monodelphis domestica* Dbx1: XP_001368121.1; Dbx2: XP_001375030.1), chicken (*Gallus gallus* Dbx1: NP_001186403.1; Dbx2: NP_001263283.1), python (*Python bivittatus* Dbx1: XP_007425354; Dbx2: XP_007424949.1), turtle (*Chrysemys picta bellii* Dbx1: XP_005305394; Dbx2: XP_005298010), African frog (*Xenopus laevis* Dbx1: NP_001079210.1; Dbx2: NP_001233246.1), western frog (*Xenopus tropicalis* Dbx1: XP_002940015; Dbx2: XP_002932867.1), coelacanth (*Latimeria chalumnae* Dbx1: XP_005997347.1; Dbx2: XP_006012514.1), zebrafish (*Danio rerio* Dbx1b: NP_571253; Dbx2: BC091853), sea squirt (*Ciona intestinalis* KH2012:KH.C3.142), amphioxus (*Branchiostoma floridae* XP_002608529), acorn worm (*Saccoglossus kowalevskii* NP_001158370.1), sea urchin (*Strongylocentrotus purpuratus* XP_001198056.2), fruit fly (*Drosophila melanogaster* NP_647677.2), jewel wasp (*Nasonia vitripennis* XP_001599133.1), lingula (*Lingula anatina* XP_013413513.1), marine annelid (*Platynereis dumerilii* SAP35630.1).

Dbx protein sequences from little skate (*Leucoraja erinacea*), lamprey (*Petromyzon marinus*), acorn worm (*Ptychodera flava*), sea bat (*Patiria miniata*), sea snail (*Lottia giganta*), octopus (*Octopus bimaculoides*), annelid worm (*Capitella teleta*), water flea (*Daphnia pulex*) and centipede (*Strigamia maritima*) were recovered and manually reconstructed from NCBI [23], Ensembl [27] or UCSC [28]. The Dbx sequence of the sea squirt *Ciona intestinalis* was further confirmed by screening a cDNA library kindly provided by Dr. M. Branno (Stazione Zoologica A. Dohrn, Napoli, Italy). Despite reiterated analyses (by BLAST and BLASTp of several Dbx sequences), we were unable to find *Dbx*-related sequences in all nematode genomes available in the UCSC database [28].

Protein sequences were aligned using the MUSCLE 3.6 software [29], and the resulting alignment was manually improved. A phylogenetic reconstruction was computed online [30] with a maximum likelihood algorithm [31] using a WAG substitution model matrix and 4 gamma categories, with a shape parameter estimated to 1.043. Statistical support was assessed using aLRT [32]. The software iTOL [33] was used to draw the phylogenetic tree.

The protein secondary structure was predicted by the software SABLE [34], using the Sable II server with the wApproximator available algorithm. The relative surface accessibility (RSA) was computed by the software NetSUrfP [35].

### DNA constructs

All constructs were generated by standard cloning procedures using restriction enzymes (New England Biolabs), T4 DNA ligase (Invitrogen), Shrimp alkaline phosphatase (Invitrogen) and Phusion polymerase (New England Biolabs) and produced using an Endo-Free Maxi prep kit (Qiagen).

For luciferase assays, the following vectors [16] were used: pMH100-hsp-TK-luc2 (reporter plasmid containing five copies of the DNA-binding site for Gal4 (UAS) upstream of the firefly luciferase gene), pKW2T-mGrg4 (Groucho expression vector) and pRL-Renilla (Renilla luciferase plasmid). Plasmids encoding the Gal4 DNA-binding domain (DB) fused to Dbx1 domains were generated by PCR; inserts were cloned into pCMX-Gal4 [16] following NheI/EcoRI digestion. Dbx1 domains correspond to amino acids (aa) 36–50 (RD1), 105–127 (RD2) and 311–335 (Cter) of the mouse protein. The VP16 transcriptional activator and Engrailed repressor domain (EnRD) fused to Nkx6.1 HD and Gal4 DB were used as positive and negative controls, respectively [16]. All of these but Dbx1 constructs were kindly provided by Prof. J. Ericson (The Karolinska Institute, Stockholm, Sweden).

For *in ovo* electroporation experiments, constructs were made in a pCAGG-IRES-EGFP plasmid. All subcloned sequences were preceded by a Kozak consensus sequence and an HA-epitope tag (YPYDVPDYA). The *Ciona intestinalis* and *Danio rerio* cDNAs were obtained by RT-PCR from cDNA libraries kindly provided by Dr. M. Branno (Stazione Zoologica A. Dohrn, Napoli, Italy) and Dr. S. Schneider-Maunoury (Institut de Biologie Paris-Seine, Paris, France), respectively. The *Saccoglossus kowalevskii* cDNA was kindly provided by Prof. C. Lowe (Hopkins Marine Station, Stanford University, CA, USA). Deletion mutants of mDbx1 were obtained by removing sequences encoding residues 36–50 (∆RD1), 105–127 (∆RD2) and 311–335 (∆Cter). The sDbx∆Cter construct was generated by removing the last 21 aa of the *Saccoglossus* sequence, while the ciDbx + Cter was generated by adding the last 25 aa of the mDbx1 to the *Ciona* sequence.

### Luciferase assays

COS-7 cells were cultured in DMEM containing Glutamax and supplemented with 10 % fetal calf serum, 100 IU/mL penicillin and 100 µg/mL streptomycin (all from Invitrogen). Cells were seeded at approximately 20 % confluence in a 96-well culture plate. They were transfected 24 h later (at ~50 % confluence) and harvested the following day (~80 % confluence). Transfection was performed using Lipofectamine 2000 (Invitrogen) according to the manufacturer’s instructions using 25 ng of pMH100-hsp-TK-luc2, 10 ng of pRL-Renilla, 75 ng of pKW2T-mGrg4 (or empty vector) and 50 ng of the Gal4-Dbx1 domains constructs.

The “Dual Luciferase Assay kit” (Promega) was used to measure firefly and Renilla luciferase activities sequentially using a TriStar LB 941 luminometer (Berthold Technologies). However, we found the Renilla luciferase activity to be significantly modulated by the different Gal4 fusion proteins. This was also observed using another reporter plasmid encoding β-galactosidase. We therefore decided to normalize the firefly luminescence obtained for each Gal4 construct by that of Gal4 alone. For each condition, measurements were taken on 10–15 wells obtained from at least three independent experiments. Experimental and control conditions were compared using Kolmogorov–Smirnov and Mann–Whitney nonparametric tests.

### *In ovo* electroporation

Fertilized chick eggs were obtained from Les Bruyères (Dangers, France). DNA solutions (2 μg/μL, 0.01 % fastgreen) were injected into the spinal cord of HH10–12 embryos [36] using a stretched glass capillary. DNA was electroporated (5 pulses of 25 V and 50 ms at 10 Hz) using a CUY21 electroporator and CUY611p7-2 electrodes (Nepagene, Chiba, Japan). Embryos were collected after 30–42 h at 39 °C (HH21–24), fixed for 1 h in 4 % paraformaldehyde, PBS at 4 °C, cryoprotected overnight in 30 % sucrose, PBS at 4 °C, and embedded in OCT compound (Sakura). Twenty-μm-thick sections were obtained using a Leica CM3050 cryostat. For immunostaining, cryosections were incubated in 0.1 % Triton, PBS supplemented with 1 % horse serum or 0.1 % BSA as blocking reagents. The following primary antibodies were applied on sections overnight at 4 °C: rabbit anti-GFP (Invitrogen, 1:1000), mouse anti-Evx1/2 ([37], 1:50) and mouse anti-En1 ([37], 1:30). After five washes, slices were incubated with secondary antibodies coupled to Alexa488 (Invitrogen) or Cy3 (Jackson ImmunoResearch) and DAPI for 30 min to one hour at room temperature. Slices were then mounted in Vectashield (Vector), and images were acquired using a Leica SP5 or Zeiss LSM710 confocal microscope. Quantifications were performed on sections collected from a minimum of three embryos per condition; the precise number of sections considered for each condition is indicated on the figures. Statistical analysis was performed by comparing the number of Evx1/2^+^ or En1^+^ cells on each side of the spinal cord using paired Student’s *t* test.

## Results

### *In silico* identification of Dbx1 and 2 protein domains

**Fig. 1:**
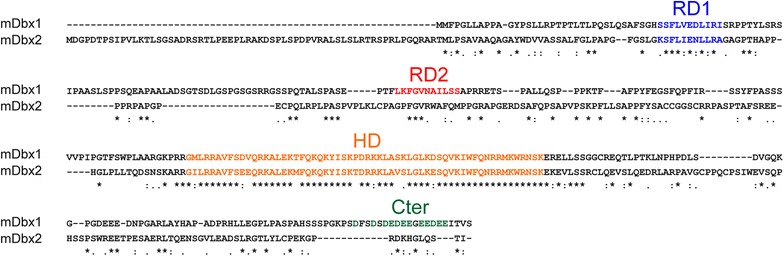
Sequence analysis of mouse Dbx1 and Dbx2 proteins. Protein sequence alignment of mouse Dbx1 and Dbx2. The functional and hypothetical domains are highlighted in *blue* (RD1), *red* (RD2), *orange* (HD) and *green* (Cter). Conservation of residues is indicated below according to the nomenclature of the ClustalX software

To identify conserved protein domains in Dbx proteins, we first aligned the mouse Dbx1 and Dbx2 proteins. This showed that the two sequences share an identity of ~40 %, mainly due to the HD (Fig. 1). Muhr and colleagues [16] identified the endecapeptides LKFGVNAILSS and KSFLIENLLRA as Engrailed Homology 1 (eh1)-like putative repressor domains for Dbx1 and Dbx2, respectively (Fig. 1, red and blue, respectively). In addition, Ma et al. [5] reported that in *Xenopus*, the eh1-like domain of Dbx2 aligns with a sequence of Dbx1 that is distinct from the one identified by Muhr and colleagues [16], suggesting the existence of two repressor domains in Dbx1 and only one in Dbx2. In order to avoid confusion, we subsequently refer to RD1 for the sequence that appears conserved between Dbx1 and Dbx2 (and proposed to behave as a transcriptional repressor in Dbx2 [16]) and RD2 for the sequence that is specific to Dbx1 (and previously suggested to be a putative repressor domain [16]).

In the carboxy-terminal region (Cter) of Dbx1, we observed an acidic residues-rich domain: DEDEEGEEDEE (Fig. 1, green). Acidic domains, also known as “acid blob” or “negative noodles” [38], were originally identified in the lambda repressor of bacteria as being involved in transcriptional activation [39, 40]. Furthermore, such hydrophilic regions were reported to be a characteristic of transcription factors that positively regulate transcription in eukaryotes [41] and, thus, might represent a putative trans-activation domain. Mutational studies on the acidic blob showed that the motif works irrespective of a specific sequence, but must show an excess of acidic residues in a clustered or unclustered sequence [38].

**Fig. 2:**
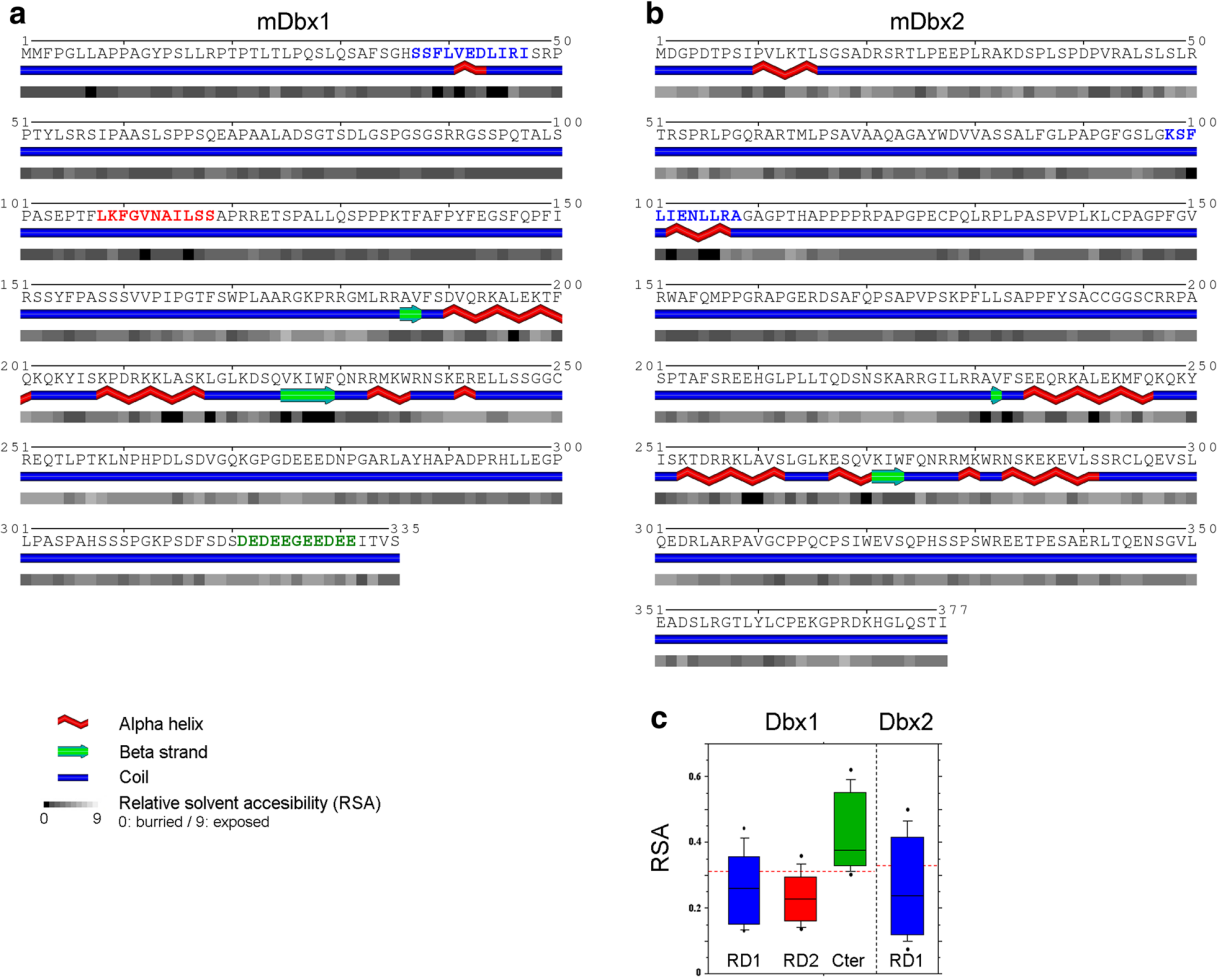
Secondary structure prediction of Dbx1 and Dbx2 proteins. Primary sequence and secondary structure prediction for mouse Dbx1 (**a**) and Dbx2 (**b**). The RD1 of Dbx1 and Dbx2 (*blue*) is predicted to be in a helix conformation with a relatively low solvent accessibility. The RD2 of Dbx1 (*red*) also has a relatively low solvent accessibility but no specific secondary structure predicted. The Cter domain of Dbx1 (*green*) has a high solvent accessibility due to the enrichment in acidic residues, and no specific secondary structure predicted. The three helices of the homeodomain are correctly predicted for both Dbx1 and Dbx2. **c** Boxplot indicating the relative solvent accessibility (RSA) of the various domains. The *dashed line* in *red* indicates the average RSA for each protein

The analysis of the predicted secondary structures of the Dbx1 (Fig. 2a) and Dbx2 (Fig. 2b) mouse proteins, in addition to the two HDs being as expected in helix-turn-helix configuration, showed that: (1) the RD1 endecapeptides were both predicted to adopt a partially helix-like conformation and characterized by having four out of 11 completely buried amino acids; (2) the RD2 domain of Dbx1 was predicted to adopt a coil conformation, with two out of 11 amino acid residues completely buried; and (3) the Cter acidic residues-rich domain was predicted to adopt a coil conformation, with none of its amino acids expected to be in a completely buried configuration (Fig. 2). This suggested that only the Cter domain was exposed to the surface of the protein.

Further analysis of the relative solvent accessibility (RSA) indicated that the average RSA values of the Dbx1 and Dbx2 RD1 domains were not significantly different (0.262 ± 0.13 and 0.264 ± 0.15, respectively; Fig. 2c). The average RSA value of the RD2 domain of Dbx1 (0.235 ± 0.08) was also not significantly different from the RD1 of either Dbx1 or Dbx2. Together, the high similarity of sequence and structure between the RD1 endecapeptides of Dbx1 and Dbx2 suggested that the two domains could play the same functional role and in order to be accessible would have to be unburied. By contrast, the average RSA value of the Cter domain (0.427 ± 0.11) was significantly higher than any of the RDs (Fig. 2c), suggesting that it is exposed at the surface of the protein and, thus, possibly represents a protein interaction domain.

### Evolution of Dbx proteins

**Fig. 3:**
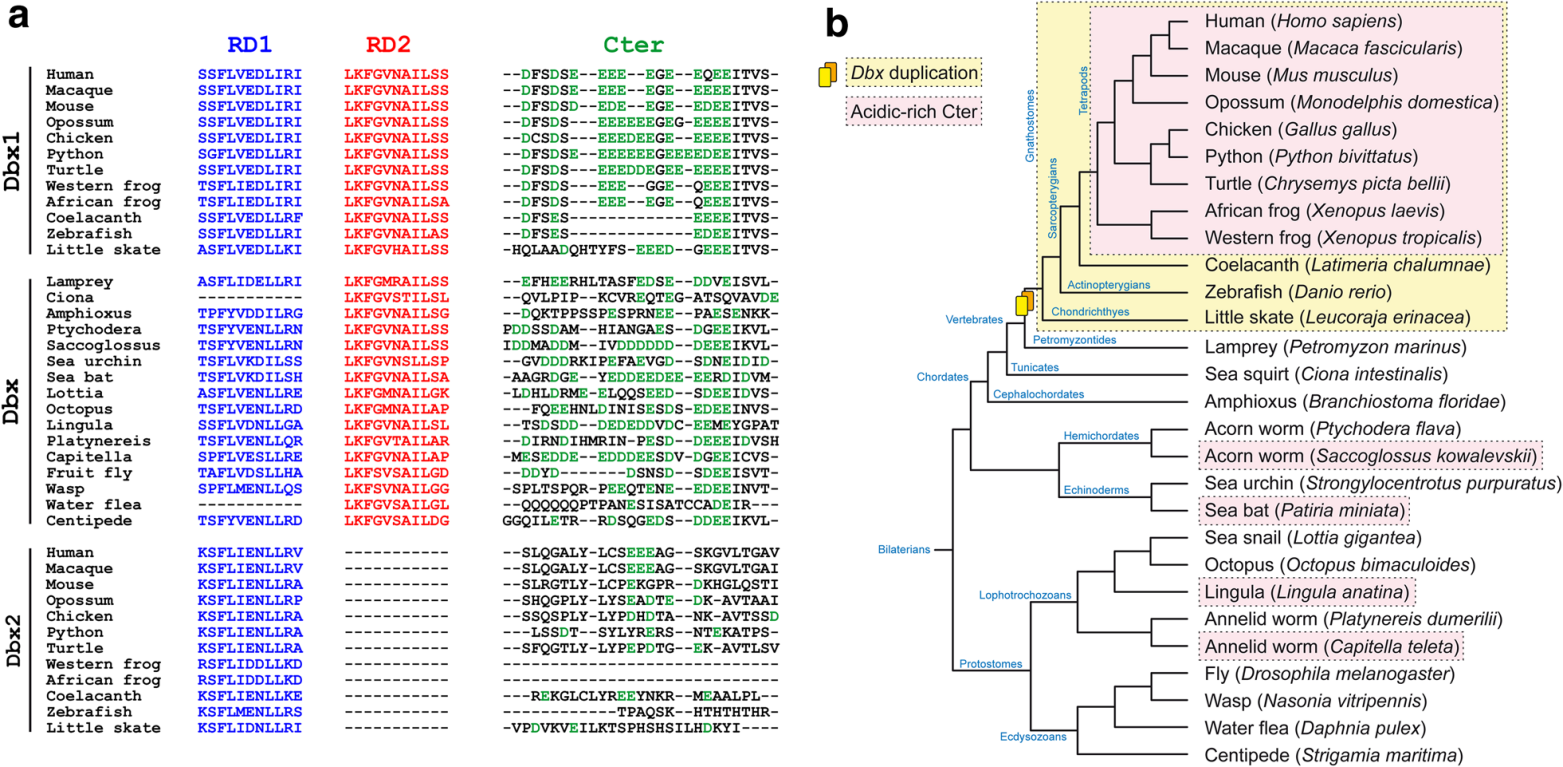
Multiple alignment of the functional domains in the Dbx protein family. **a** Multiple alignment of representative sequences of the Dbx family allowed the identification of a RD1 (*blue*) in all species with the exception of the tunicate *Ciona intestinalis* and the crustacean *Daphnia pulex*. RD2 (*red*) is found in all Dbx and Dbx1 sequences, but not in Dbx2 proteins. A specific enrichment and clusterization of acidic residues (*green*) is found at the C-terminus of Dbx1 proteins of tetrapods as well as in *Saccoglossus kowalevskii*, *Patiria miniata*, *Lingula anatina* and *Capitella teleta*. **b** Phylogenetic tree of the species used in **a**. The *orange box* indicates species containing both Dbx1 and Dbx2 sequences, suggesting that the Dbx duplication occurred in the common ancestor to all gnathostomes. *Pink boxes* indicate species in which it was possible to find a stretch of 10 amino acids containing at least 80 % of D or E within the C-terminus

To begin investigating the function of each Dbx protein domain, we analyzed their conservation during evolution by multiple alignment of protein sequences available for the Dbx family in metazoans. Protein sequences unambiguously belonging to the Dbx family were found in several protostomes, indicating that a *dbx* ancestral gene was already present in the common ancestor of all bilaterians (Fig. 3). Since *dbx1* and *dbx2* genes can be found in chondrichthyes, actinopterygians and sarcopterygians, compared to a single gene in petromyzontides, tunicates and cephalochordates, the divergence between both genes likely occurred in the gnathostomes lineage (Fig. 3b). Alternatively, if generated by a whole-genome duplication event, those two paralogs might have been present before the cyclostome/gnathostome split and lost secondarily in cyclostomes [42, 43]. In addition, the supplementary whole-genome duplication in teleost resulted in the presence of two Dbx1 paralogs, namely Dbx1a and Dbx1b. For greater clarity, only the latter was used when considering teleosts since it shows a slightly higher identity with mouse Dbx1 [6]. A multiple alignment of Dbx proteins selected in order to have a significant, although not exhaustive, representation of metazoan organisms, is available (see Additional file 1).

Interestingly, the RD1, RD2 and Cter domains found in mouse Dbx1 were differentially conserved among species. The RD1 domain showed a high conservation level in all proteins of the Dbx family, with the noticeable exception of *Ciona intestinalis* and *Daphnia pulex* Dbx representatives (Fig. 3a), suggesting a specific loss of this domain in tunicates (the sequence of *Ciona savignyi* also lacks the RD1 domain) and crustaceans. The RD2 domain was well conserved in all species but, as already observed in mouse (Fig. 1) and Xenopus [5], it was absent in all Dbx2 sequences (Fig. 3a), indicating that a loss of this domain most probably occurred very soon after the divergence between Dbx1 and Dbx2. The evolution of the Cter domain appeared more complex as the alignment did not allow us to simply discriminate between the presence or absence of the domain, but rather gave us indications on the enrichment and clusterization of acidic residues. For better clarity, we decided to subsequently refer to species bearing a Cter as those in which it was possible to identify a stretch of ten amino acids containing at least 80 % Asp or Glu residues. We found Dbx1 proteins from all tetrapods as well as Dbx sequences from *Saccoglossus kowalevskii*, *Patiria miniata*, *Lingula anatina* and *Capitella teleta* to match such a criteria (Fig. 3). By contrast, Dbx2 sequences did not display any specific enrichment in acidic residues at the C-terminus (Fig. 3a).

We performed a maximum likelihood phylogenetic reconstruction of the Dbx protein family. As indicated in Additional file 2, Dbx and Dbx1 sequences of all invertebrates and vertebrates were grouped together with a strong statistical support. A second clade grouped all the Dbx2 sequences of vertebrates. This result can be easily correlated with the various modifications and losses that have been sustained by Dbx2 proteins (i.e., presence/absence of RD2 and Cter domains).

### Transcriptional activity of Dbx1 domains in vitro

**Fig. 4:**
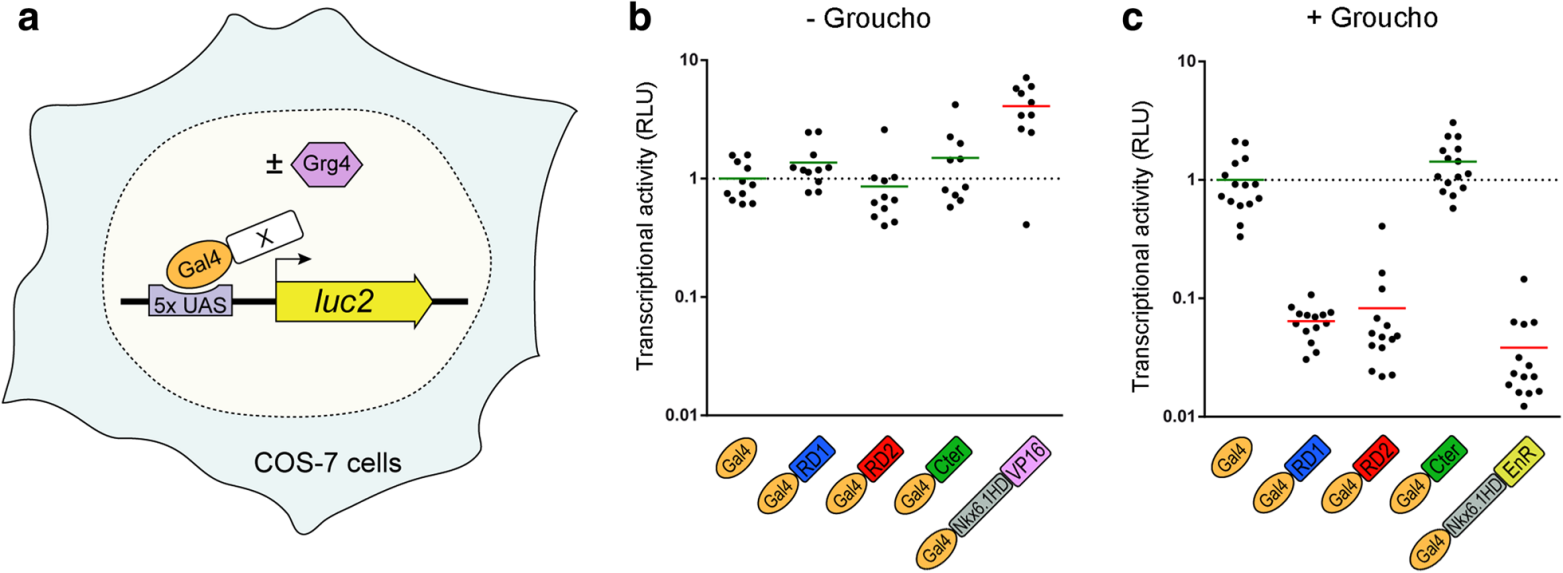
Transcriptional activity of Dbx1 domains. **a** Experimental design of the luciferase assay allowing to assess the transcriptional activity of Gal4-fused domains of mouse Dbx1. **b**, **c** Quantification of the luciferase signal in the absence (**b**) or presence (**c**) of Groucho. Each *dot* represents one well of transfected cells. Values were normalized to 1 in control (Gal4-only); the *dashed line marks* the baseline of luciferase expression. VP16 and Engrailed repressor domain (EnR) fused to Nkx6.1 HD were used as positive and negative controls, respectively. The means are depicted by a *horizontal bar*, *red* indicates a significant difference compared to control (*p* < 0.01 using both Kolmogorov–Smirnov and Mann–Whitney tests), and *green* indicates no significant difference. Both RDs behave as Groucho-dependent transcriptional repressors, whereas the Cter domain has no transcriptional activity in this assay

Out of the two putative eh1-like domains of Dbx1, namely RD1 and RD2, only the latter had been shown to interact with Groucho in a GST pull-down assay [16]. Although this suggests that Dbx1 behaves as a repressor through its RD2 domain, the intrinsic transcriptional activity of both RD1 and RD2 remains to be established, as it is the case for the Cter domain. We therefore decided to test the ability of each of the Dbx1 domains to activate or repress transcription by performing in vitro luciferase assay (Fig. 4a). The RD1, RD2 and Cter domains of mouse Dbx1 were fused to the Gal4 DNA-binding domain. These constructs were co-transfected in COS-7 cells together with a reporter in which luciferase expression is driven by a thymidine kinase promoter that can be modulated by Gal4-dependent upstream acting sequences (UAS). As the activity of the eh1 repression domain is mediated by interaction with Groucho co-repressors [16], we expressed our constructs in the absence or presence of mouse Groucho4 (mGrg4). We verified that the reporter could respond to both repressor and activator proteins using constructs expressing a strong repressor (Gal4-Nkx6.1HD fused to the Engrailed Repression (EnR) domain) or a strong activator (Gal4-Nkx6.1HD fused to the VP16 activation domain) [16]. As expected, we found the VP16 construct able to increase luciferase expression in the absence of Groucho (by ~fourfold compared to Gal4 alone; Fig. 4b), whereas the EnR construct robustly repressed reporter expression in the presence of Groucho (by more than tenfold; Fig. 4c).

Neither RD1 nor RD2 domain had a significant effect on luciferase expression when expressed in the absence of Groucho (Fig. 4b). By contrast, both RDs were able to repress reporter activity by ~tenfold in its presence (Fig. 4c), indicating that these domains are *bona fide* Groucho-dependent transcriptional repressors. The Cter domain remained unable to significantly modulate luciferase expression regardless of the presence of Groucho (Fig. 4b, c), indicating that this domain is devoid of transcriptional activity when isolated from the rest of the Dbx1 protein.

### Function of Dbx1 RD domains in vivo

In the ventral spinal cord, Dbx1 is expressed in the most dorsal (p0) progenitor domain [37] and establishes the distinction between p0 and p1 progenitor domains. In Dbx1 null mutant mice [10], p0 progenitors fail to generate v0 interneurons (identified by the expression of Evx1/2) and instead give rise to v1 interneurons (expressing En1). Conversely, overexpression of the wild-type mouse Dbx1 protein (mDbx1) in the chick developing spinal cord is sufficient to induce the generation of v0 interneurons and prevent the production of v1 interneurons [10].

**Fig. 5:**
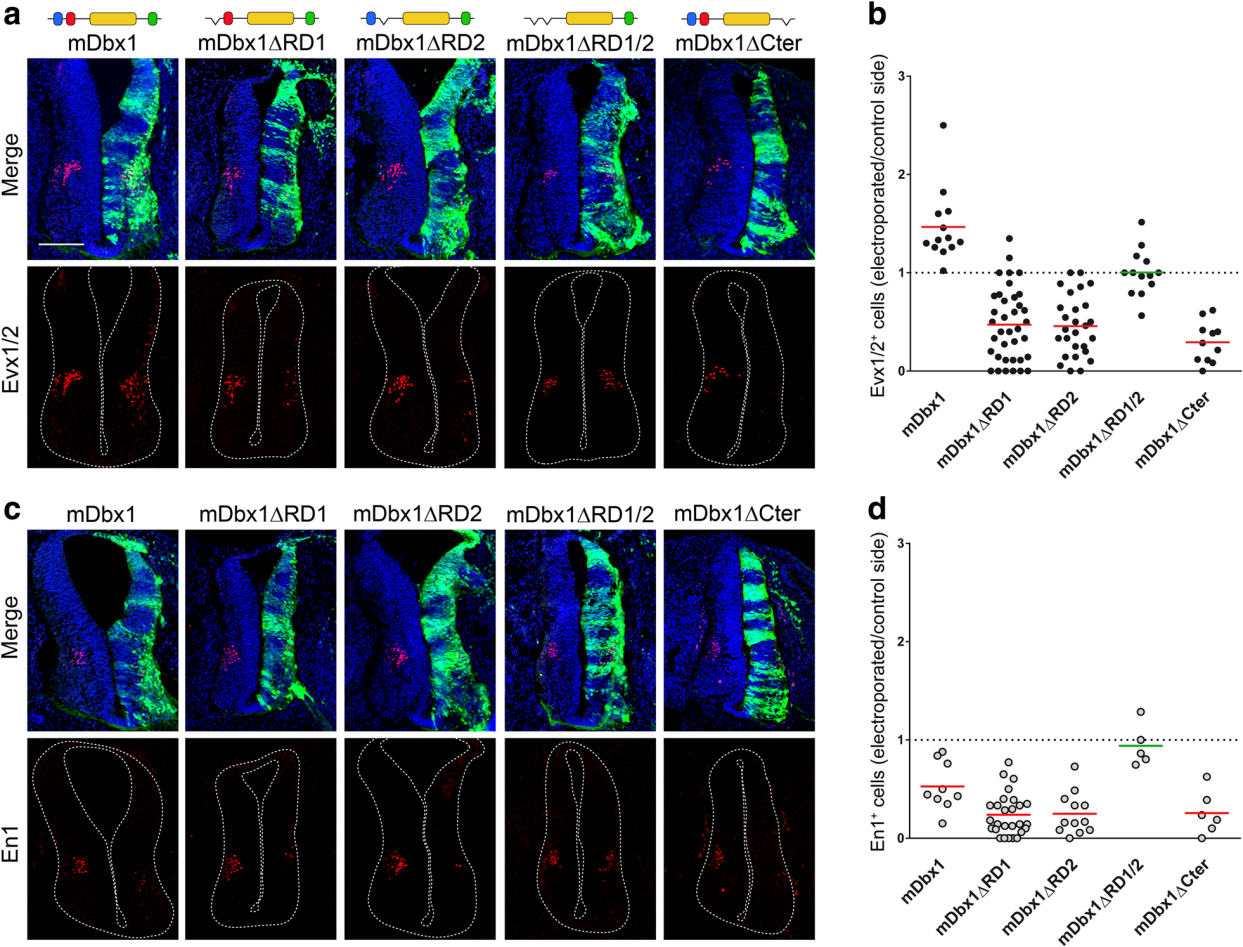
Function of Dbx1 domains in vivo. **a**, **c** Cryosections of chick spinal cord electroporated with plasmids encoding mDbx1, mDbx1∆RD1, mDbx1∆RD2, mDbx1∆RD1/2 or mDbx1∆Cter and subjected to DAPI (*blue*), GFP (*green*) and either Evx1/2 (**a**) or En1 (**c**) labeling (*red*). GFP allowed to visualize the electroporated side. **b**, **d** Quantifications of Evx1/2^+^ (**b**) or En1^+^ (**d**) cells on the electroporated side relative to the control side are indicated. *Each dot* represents one section, the *horizontal bar* corresponds to the average for each condition, and statistical significance is color coded (*red* *p* < 0.01 using paired Student’s *t* test; *green* nonsignificant). *Scale bar* 100 µm

To gain insight into the specific function of each of the Dbx1 eh1-like motifs in vivo, we generated constructs encoding the mouse protein lacking either one of the two RDs (mDbx1∆RD1 and mDbx1∆RD2) and expressed them in the chick embryo neural tube by *in ovo* electroporation. Only one side of the neural tube was targeted, allowing comparison with the non-electroporated (control) side. Electroporated cells and transfection efficiency were visualized through the bicistronic expression of GFP. We monitored the ability of wild-type and RD-deleted Dbx1 proteins to modulate cell fate specification by immunostaining using Evx1/2 and En1 antibodies. As previously reported [10], full-length mDbx1 promoted Evx1/2^+^ interneurons fate at the expense of En1^+^ interneurons (Fig. 5). When either RD1 or RD2 was removed, we found Evx1/2^+^ interneuron differentiation to be inhibited compared to the non-electroporated side of the spinal cord (Fig. 5a, b), indicating that (1) both repressor domains are strictly required for v0 fate specification and (2) removal of either one of them unravels an inhibitory activity for the deleted protein. By contrast, no significant difference in the capacity of the mDbx1∆RD1 and mDbx1∆RD2 to inhibit En1^+^ interneurons differentiation was observed compared to full-length mDbx1 (Fig. 5c, d), indicating that both repressor domains of Dbx1 are individually dispensable to prevent v1 fate.

In addition, we found that a Dbx1 construct lacking both its repressor domains (mDbx1∆RD1/2) was unable to promote or inhibit the generation of either Evx1/2^+^ or En1^+^ interneurons (Fig. 5). These data show that the two RDs are functionally active, confirming that the RD2 domain functions as a repressor as previously suggested [16] and showing that the RD1 has a similar activity. They also show that both aspects of the fate specification activity of Dbx1 (v0 induction and v1 inhibition) are mediated by transcriptional repression. However, if RD1 and RD2 display redundant function with respect to inhibition of v1 generation, both are required for the induction of v0 fate.

### Function of Dbx1 Cter domain in vivo

Despite the absence of intrinsic transcriptional activity of the Dbx1 Cter domain in vitro, its strong conservation among tetrapods prompted us to investigate whether it has a role in vivo in the context of the full protein. We generated a construct encoding a mouse Dbx1 protein lacking its Cter domain (mDbx1∆Cter) and tested its activity by *in ovo* electroporation. Opposite to the full-length mDbx1, we found the mDbx1∆Cter protein to prevent the generation of Evx1/2^+^ interneurons (Fig. 5a, b). Incidentally, as it is the case for each of the two repressor domains, the removal of the Cter revealed an inhibitory activity for the rest of the protein on v0 generation. By contrast, removal of the Cter had no significant effect on the ability of Dbx1 to block En1^+^ interneurons generation (Fig. 5c, d), indicating that it is dispensable for v1 fate inhibition. The Cter domain therefore appears required for Dbx1-induced v0 fate specification in the vertebrate spinal cord and possibly functions by preventing a repressor activity of the protein. Furthermore, these results also show that each RD and Cter domains of Dbx1 are absolutely necessary for promoting v0 fate, suggesting distinct mechanisms mediating Dbx1-dependent v0 fate induction and v1 inhibition.

### In vivo activity of Dbx proteins from different species

In order to better understand how the evolutionary acquisition or loss of functional domains may have conferred specific activities to Dbx proteins, we tested the consequences of an overexpression of Dbx from different species on the production of v0 and v1 interneurons by *in ovo* electroporation in the chick embryo neural tube (Additional file 3). We decided to use: (1) Dbx from *Ciona intestinalis* (ciDbx) as a mDbx1 ortholog displaying a quite different composition in terms of domains (lacking both RD1 and Cter domains); (2) zebrafish (*Danio rerio*) Dbx1b (zDbx1b) as a closer ortholog but devoid of Cter domain (we favored zDbx1b over zDbx1a due to a slightly better conservation of the RD1 and RD2 domains with respect to the mouse protein); and (3) *Saccoglossus kowalevskii* Dbx (sDbx) as an ortholog from a distant specie but yet retaining a structure close to that of mDbx1 (i.e., bearing RD1, RD2 and Cter domains).

**Fig. 6:**
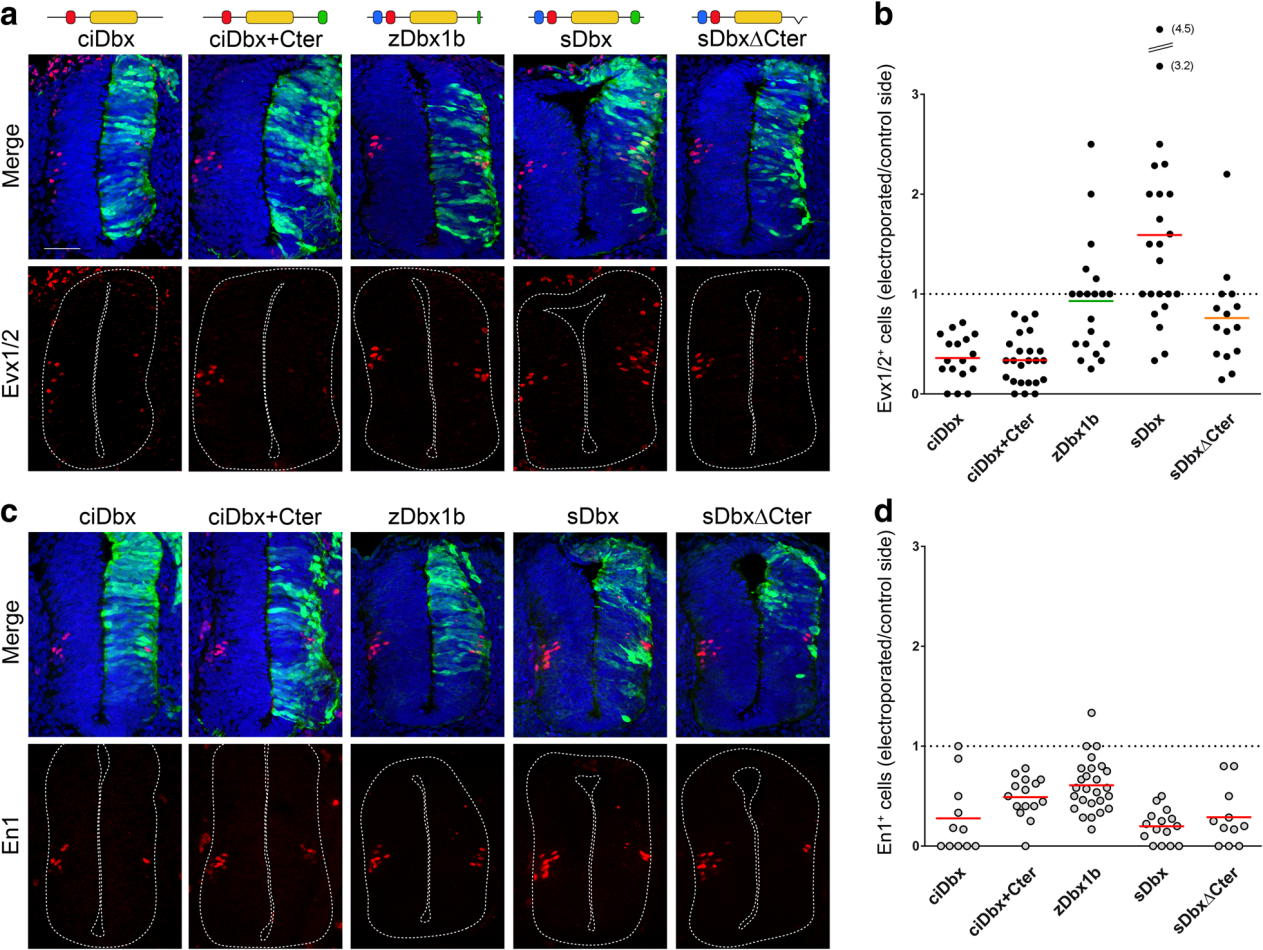
In vivo function of Dbx1 from different species. *In ovo* electroporation experiments using plasmids encoding Dbx homologs from *Ciona* (ciDbx), *Saccoglossus* (sDbx), Zebrafish (zDbx1b) as well as Cter-modified versions (ciDbx + Cter and sDbx∆Cter). Spinal cord cryosections were immunostained for Evx1/2 (**a**) or En1 (**c**) (*red*), DAPI (*blue*) and GFP (*green*) to visualize the electroporated side. **b**, **d** Quantifications of Evx1/2^+^ (**b**) or En1^+^ (**d**) cells on the electroporated side relative to the control side are indicated. *Each dot* represents one section, the *horizontal bar* corresponds to the average for each condition, and statistical significance is *color* *coded* (*red* *p* < 0.01 using paired Student’s *t* test; *orange* *p* < 0.05; *green* nonsignificant). *Scale bar* 50 µm

The ciDbx protein strongly inhibited the generation of both v0 and v1 interneurons (Fig. 6), reminiscent of the mDbx1 truncation mutants lacking either RDs or Cter domains. We next assessed whether adding the mouse Cter domain to the *Ciona* protein could promote v0 generation. However, a ciDbx + Cter mutant protein had no measurable effect on the activity of ciDbx (Fig. 6), indicating that this domain is not sufficient, in the context of the *Ciona* protein, to confer v0 fate determination properties. Since ciDbx also lacks the RD1 domain, we investigated what was the activity of Dbx proteins naturally lacking only the Cter. We therefore tested the zDbx1b which bears highly conserved RD1 and RD2 but displays a very short cluster of acidic residues at the C-terminus (Fig. 3a). We found zDbx1b able to inhibit the generation of En1^+^ interneurons (Fig. 6c, d) but unable to promote or inhibit that of Evx1/2^+^ interneurons (Fig. 6a, b), thus behaving as the mDbx1∆RD1/2 construct. This strongly suggested that the lack of RD1 in the ciDbx protein is unlikely to be responsible for the absence of Evx1/2^+^-inducing activity but rather that either the Cter alone or in synergism with the RD1 is. Interestingly, the *Saccoglossus kowalewskii* Dbx protein (sDbx) bears a fairly conserved similarity in amino acids sequence in both the RD1 and Cter domain compared to the mouse protein. Indeed, when electroporated in the chick neural tube, sDbx promoted the differentiation of Evx1/2^+^ interneurons in the same extent as mDbx1 (but with increased variability; Fig. 6a, b) and decreased En1^+^ interneurons numbers (Fig. 6c, d). Evx1/2^+^ interneuron fate-inducing activity was abolished upon removal of the Cter domain (Fig. 6a, b). We thus confirmed that the Cter domain is required for the promotion of v0 fate, consistent with our previous observations using mDbx1 constructs.

Taken together with our electroporation experiments using mDbx1 deletion constructs, these results indicate that domain composition of Dbx family proteins is critical for the promotion of v0 fate. Furthermore, we found a striking correlation between the ability of Dbx proteins to induce Evx1/2^+^ interneurons and the enrichment and clusterization of acidic residues at the C-terminus.

## Discussion

The vertebrate spinal cord has been used as a model to understand the genetic mechanisms that underlie neuronal diversity [17]. The combinatorial expression of TFs defines the identity of discrete progenitor domains within the ventral spinal cord [44]. These progenitors then give rise to distinct classes of ventral neurons (v0–v3 interneurons and motoneurons). The appropriate production of neuronal subtypes therefore relies on the expression domains and transcriptional activity/specificity of TFs within progenitors. Cross-repression between TFs allows the formation of sharp boundaries between progenitor domains and has been shown to be mediated by a repressive activity of these HD proteins. Indeed, most of them are transcriptional repressors that possess an Engrailed homology-1 (eh1) domain able to recruit the co-repressor Groucho [16].

Dbx1 is a HD TF playing crucial roles in dorsoventral patterning of the spinal cord. It promotes v0/Evx1^+^ and inhibits v1/En1^+^ interneurons fates [10]. Until now, both functions were thought to be coupled, as Dbx1 and Evx1 gain- and loss-of-function was shown to prevent and activate En1 expression, respectively [10, 45], whereas Prdm12, a gene expressed in the p1 progenitor domain, had opposite effect through cross-repressive interactions with Dbx1 [46]. In addition, the precise contribution of Dbx1 conserved domains to its biological activity remained elusive: Although Muhr et al. [16] showed that Dbx1 RD2 binds Groucho, they did not assess the consequences of such an interaction. Our results make the picture more complex and interesting from an evolutionary point of view. Luciferase assays and *in ovo* electroporation experiments demonstrated that both RD1 and RD2 are genuine Groucho-dependent repressor domains. RD1 and RD2 are individually dispensable for the inhibition of alternative v1 cell fate in vivo, suggesting that they play redundant roles. By contrast, they appear to be both required, suggesting that they act synergistically, for the induction of v0 fate. These observations also argue that distinct molecular pathways underlie these two activities.

Sequence analysis of the Dbx family also allowed us to identify a novel Cter domain enriched in acidic residues. Such domains were reported to be in two possible conformations: unclustered or clustered [38]. Although similar protein domains were found to mediate transcriptional activation in both prokaryotes and eukaryotes [38, 41], the Cter domain of mDbx1 showed no intrinsic transcriptional activity when isolated from the rest of the protein. However, both mouse and *Saccoglossus* Cter domains are absolutely necessary in vivo in the induction of v0 fate. In addition, their removal uncovers a strong inhibitory activity of the protein on v0 fate, as do mutations of each RD. Transcriptional repression is also required for both induction of v0 and inhibition of v1 cell fates, as shown by the ∆RD1/∆RD2 mutant and consistent with the idea that most biological activity mediated by Dbx1 occurs through repression of target genes. We can, thus, speculate that the Cter domain modulates transcriptional repression mediated by the RDs or is involved in target selectivity, these two hypotheses being non-mutually exclusive.

The Dbx1 protein domains showed not only different functions, but also different evolutionary histories. In fact, while the RD1 displayed a quite well-conserved structure among the great majority of bilaterians analyzed so far, the Cter domain seems to have sustained different evolutionary histories among bilaterians. Given the phylogenetic relationship between tetrapods, hemichordates, echinoderms and lophotrochozoans, two hypotheses regarding the evolution of the Cter domain can be proposed: (1) the Cter domain was already present in the common ancestor to all bilaterians and secondary losses occurred in multiple lineages or (2) convergent evolution drove a progressive enrichment and clusterization of acidic residues at the C-terminus independently in several species. A pattern of specific losses of genes and domains has already been documented by comparing the genomes of early divergent deuterostomes and vertebrates [47, 48], arguing in favor of the first hypothesis. Nevertheless, the multiple convergent C-terminal acidic enrichment scenario is supported by: (1) the strong conservation of the Cter among tetrapods compared to its fickle preservation in other Cter-containing Dbx proteins and (2) the unparsimonious number of putative losses that would be necessary to account for the current representation of the Cter domain among bilaterians.

We found a strong correlation between the enrichment and clusterization of acidic residues in the Cter domain and the ability of Dbx proteins to modulate Evx1/2 expression. Mouse and *Saccoglossus* proteins harbor a long clustered Cter domain (~10 acidic residues) and reproducibly induce Evx1/2 expression in the chick spinal cord. At the other end of the spectrum, proteins showing no specific acidic enrichment at the C-terminus, whether naturally (ciDbx) or artificially (∆Cter mutants), robustly inhibit Evx1/2^+^ fate. Zebrafish Dbx1b displays a short cluster of acidic residues, as other teleost fishes, and was found able to neither promote nor repress Evx1/2^+^ interneuron fate. Since the mouse and zebrafish Dbx1 proteins have very well-conserved RD1, RD2 and HD, our results strongly suggest that differences in their respective activities lie in the Cter domain, although we cannot formally exclude that other less conserved regions are also involved. Our work therefore points to the idea that the evolutionary enrichment and clustering of acidic residues in the Cter domain could have been an important step in the expansion of gene regulatory networks controlling neuronal diversity, and ultimately nervous system complexity.

Such a hypothesis is reminiscent of the HD TF Ubx, whose domain composition was previously linked with the evolution of patterning in arthropods [49, 50]. *Drosophila* Ubx has the ability to prevent limb development in thoracic segments through the transcriptional repression of *dll* [51]. In velvet worms, which bear limbs on all segments, Ubx is expressed in some of the segments. When Ubx from velvet worms is expressed in *Drosophila* embryos, it is unable to repress both *dll* and limb development. The ability of *Drosophila* Ubx to repress *dll* is due to a short C-terminal alanine rich domain that is absent in velvet worms. Thus, the acquisition of a new domain within Ubx has allowed the repression of appendage development on abdominal segments in insects.

The implications of the presence of an acidic-rich Cter domain in invertebrates remain an open question that will require comparative functional studies in these organisms. In mouse, the critical function of Dbx1 in controlling the fate of neurons essential for both locomotion [10–12] and breathing [13, 14], together with the high conservation of the Cter domain among tetrapods, raises the question of the contribution of this domain to nervous system complexification associated with the adaptation to terrestrial life.

## Conclusions

We have identified a novel acidic-rich C-terminal domain within the Dbx1 transcription factor that is conserved among tetrapods as well as several invertebrates. We have shown that this domain is required for Dbx1-induced neuronal fate specification in the developing spinal cord. Our data are consistent with the idea that the acquisition of this domain during evolution is linked to increased neuronal diversity and nervous system complexity.

## Additional files

10.1186/s13227-016-0055-5 Full alignment of Dbx family proteins. Protein sequence alignment of all Dbx sequences used in this study obtained using the MUSCLE 3.6 software and manually improved. The functional and hypothetical domains are located at the following positions: RD1: 159-169, RD2: 538-548, HD: 824-883 and Cter: 1117-1134.
10.1186/s13227-016-0055-5 Maximum likelihood phylogenetic tree of the Dbx family proteins. Dbx, Dbx1 and Dbx2 sequences are indicated in pink, orange and blue, respectively. aLRT statistical support is color coded (from 70 % in green to 100 % in red). A schematic representation of the functional domains found in each sequence is also reported. The scale bar indicates the branch length that corresponds to the number of substitutions per residue.
10.1186/s13227-016-0055-5 Diagram of the electroporation experiments. (A) DNA is injected in the neural tube of developing chick embryos and transfected through electroporation in only one half of the spinal cord. (B) On the control side of the spinal cord, Dbx1 is expressed by p0 progenitors; it favors their differentiation in v0 (Evx1/2^+^) interneurons and prevents v1 (En1^+^) fate. On the electroporated side, the fate specification properties of various Dbx1 constructs are assessed by counting the number of v0 and v1 neurons generated relative to the control side.
