# Supplementary material for: Neuronal fate specification by the Dbx1 transcription factor is linked to the evolutionary acquisition of a novel functional domain
Source: EvoDevo. 2016 Aug 12;7:18. doi: 10.1186/s13227-016-0055-5 (PMC4983035; doi:10.1186/s13227-016-0055-5)
Supplement: Supplementary file 1 — 10.1186/s13227-016-0055-5 Full alignment of Dbx family proteins. Protein sequence alignment of all Dbx sequences used in this study obtained using the MUSCLE 3.6 software and manually improved. The functional and hypothetical domains are located at the following positions: RD1: 159-169, RD2: 538-548, HD: 824-883 and Cter: 1117-1134. [file 13227_2016_55_MOESM1_ESM.rtf]

                              10        20        30        40        50        60        70        80        90       100       110       120       130       140       150                       
                     ....|....|....|....|....|....|....|....|....|....|....|....|....|....|....|....|....|....|....|....|....|....|....|....|....|....|....|....|....|....|
Homsap-Dbx1          -----------------------------------------------------------------MMFP-GLL-APPAGYPSLLR-----------------------PTPTLTLP-QSLQ----------------------------- 
Macfas-Dbx1          -----------------------------------------------------------------MMFP-GLL-APPAGYPSLLR-----------------------PTPTLTLP-QSLQ----------------------------- 
Musmus-Dbx1          -----------------------------------------------------------------MMFP-GLL-APPAGYPSLLR-----------------------PTPTLTLP-QSLQ----------------------------- 
Mondom-Dbx1          -----------------------------------------------------------------MMFP-SLI-APPAVYPNLLR-----------------------PTPTLTLP-QSLQ----------------------------- 
Galgal-Dbx1          -----------------------------------------------------------------MMFP-SLI-APPAVYPSLLR-----------------------PTPTLTLP-QSLQ----------------------------- 
Pytbiv-Dbx1          -----------------------------------------------------------------MMFP-SLL-APPAVYPSLLR-----------------------PTPTLTLP-QTLQ----------------------------- 
Chrpic-Dbx1          -----------------------------------------------------------------MMFP-SLI-APPAVYPNLLR-----------------------PTPTLTLP-QSLQ----------------------------- 
Xenlae-Dbx1          -----------------------------------------------------------------MMFP-SLL-APPAVYPNLLR-----------------------PTPTLTLP-QSIQ----------------------------- 
Xentro-Dbx1          -----------------------------------------------------------------MMFP-SLL-APPAVYPNLLR-----------------------PTPTLTLP-QSIQ----------------------------- 
Latcha-Dbx1          -----------------------------------------------------------------MMFP-TVI-APPAVYPSILR-----------------------PAPTLTLP-QSLQ----------------------------- 
Danrer-Dbx1b         -----------------------------------------------------------------MMLP-SVI-APPAMYPSFLR-----------------------PSSALSLP-PALQ----------------------------- 
Leueri-Dbx1          ------------------------------------------------------------------------M-APSAMYPGLLR-----------------------PTPTLTLP-QSLQ----------------------------S 
Petmar-Dbx           -----------------------------------------------------------------MMFP--SALANVALYH--HHQQQQHHQQQQQQQQQHLLHAVYRPTPTLGFP-VSAGNSSSSQQQQQQHHQQPQQHHHQHQAAAGI 
Cioint-Dbx           -----------------------------------------------------------------MAFPGPDAVKFGW--NFLLT-----------------------PEWVFQNP-AWRV----------------------------P 
Braflo-Dbx           ------------------------------------------------------------------MYQ-GVFSPVAFPQPTVFR-------------------------PTITFP-PPLQ----------------------------- 
Ptyfla-Dbx           ------------------------------------------------------------------MFH-NVL-APSPVYQNVFR-------------------------PPAAQP-PPTS----------------------------- 
Sackow-Dbx           ------------------------------------------------------------------MFH-NVL-APSPVYQNVFR------------------------PPTVPQP-APAA----------------------------- 
Strpur-Dbx           -------------------------------------MSGLPHPGGLPLPHLMRRLEPRFDASDEKSSIFSGVLAPTPIYQGIFR--QHLAATAPQPISNTASSPQATPPPVPQQP-PAGG----------------------------- 
Patmin-Dbx           -------------MTTVDARSLPGEPPYTYAPASSIATLHAQHSLHG-VPHLLCRLD-R-DRGEDK-PPFGGLLAPSPLYHTVFRPQAFRVPMSGAGSLQQPRQQQQQQQHHHHQP-QQQQQQQANNHHPQNHHHQHHHHHHQAHQQQPA 
Lotgig-Dbx           ----------------------------------------------------------MTVNNYFGMFP-NII-APSPIYQSLFR-----------------------------APILHQG----------------------------- 
Octbim-Dbx           ---------------------------------------------------------------------------------------------------------------------------------------------PPPPPPPPP 
Linana-Dbx           ------------------------------------------------------------------MFQNSIL-----------------------------------VKPASSPPAGQPY----------------------------- 
Pladum-Dbx           ----------------------------------------------------------------MSFLPENLMMGHPAFYGQFFR----------------------MPGSAMGPVMIPPG----------------------------- 
Captel-Dbx           -----------------------------------------------------------MYGLPKNMFTDNLL-TPSHFYPGMLR-----------------------GASGLGGP-QGPG----------------------------- 
Dromel-Dbx           ------------------------------------------------------------------------------------------------------------------------------------------------------ 
Nasvit-Dbx           ------------------------------------------------------------------MLKNLQQSAM-------------------------------------------------------------------------- 
Dappul-Dbx           ------------------------------------------------------------------MLD-VTKEEPEQVAEDSVS----------------------------------------CDPISGSSDRHHHHRHEPTII---- 
Strmar-Dbx           ------------------------------------------------------------------MFPVAQVPPPPPPLYASLR--------------------------------STPV----------------------------- 
Homsap-Dbx2          ------------------------------------------------------------------MLP-SAVAAHAGAYWDVVA-----------------------SSALLNLP-AAPG----------------------------- 
Macfas-Dbx2          ------------------------------------------------------------------MLP-RAVAAHAGAYWDVVA-----------------------SSAFLNLP-AAPG----------------------------- 
Musmus-Dbx2          MDGPDTPSIPVLK---TLSGSADRSRTLPEEPLRAKDSPLSPDPVRALSLSLRTRSPRLPGQRARTMLP-SAVAAQAGAYWDVVA-----------------------SSALFGLP--APG----------------------------- 
Mondom-Dbx2          ------------------------------------------------------------------MLP-NALSARTGVYWDIVG-----------------------SSALLD---STPG----------------------------- 
Latcha-Dbx2          ------------------------------------------------------------------MLPSAVC-------WDTPG-----------------------SSPLLHAP-LLPG----------------------------- 
Galgal-Dbx2          ------------------------------------------------------------------MLP-SAL------YWDLVG-----------------------SSALLNLP-AAPG----------------------------- 
Pytbiv-Dbx2          ------------------------------------------------------------------MLPSALA-------WHCGS-----------------------SAALLGLP-GAPG----------------------------- 
Chrpic-Dbx2          ------------------------------------------------------------------MLPTALG-------WGVAG-----------------------SSALLDLP-APPG----------------------------- 
Xenlae-Dbx2          ------------------------------------------------------------------MDP-GAL-SSAVWRWDLPR-----------------------PVAHIQSSTAPPG----------------------------- 
Xentro-Dbx2          ------------------------------------------------------------------MDP-GAL-SSTFWGWDLPR-----------------------PAAHIQSSTVPPV----------------------------- 
Danrer-Dbx2          -----------------------------------------------------------------------------------------------------------------MMM-TTAG----------------------------- 
Leueri-Dbx2          ------------------------------------------------------------------MLP-SLI-PSPSVSWQVLG------------------------------PPQLPA----------------------------- 

                             160       170       180       190       200       210       220       230       240       250       260       270       280       290       300              
                     ....|....|....|....|....|....|....|....|....|....|....|....|....|....|....|....|....|....|....|....|....|....|....|....|....|....|....|....|....|....|
Homsap-Dbx1          --SAFSGHSSFLVEDLIRISR---PPAYLPR---SVPTA-SMSPPRQGAPTALTDTGAS-DL----------------GSPGPGSRRGGSPPTAFSPASET------------------------------------------------- 
Macfas-Dbx1          --SAFSGHSSFLVEDLIRISR---PPAYLPR---SVPTS-SMSPPRQGAPTALTDTGAS-DL----------------GSPGPGSRRGGSSPTAVSPASET------------------------------------------------- 
Musmus-Dbx1          --SAFSGHSSFLVEDLIRISR---PPTYLSR---SIPAA-SLSPPSQEAPAALADSGTS-DL----------------GSPGSGSRRGSSPQTALSPASEP------------------------------------------------- 
Mondom-Dbx1          --SAFSSHSSFLVEDLIRISR---PGSYLPR---SVPQP-SLSPPTSGTTSAMTDTVTS-DL---------------VSSSASSSRRGCSPQTSASQANEA------------------------------------------------- 
Galgal-Dbx1          --SAFSSHSSFLVEDLIRISR---PGAYPPR---SAPPPSSMSPPA---SAPRTDSGTP-EL---------------PGC--TAARRICSSQ---SSGSDS------------------------------------------------- 
Pytbiv-Dbx1          -SAFSGP-SGFLVEDLLRIGR---PAGYPPRGSAPPPSLSPPVGGAGAARTDAASAADL------------------GTADSSGARTSCSSQSSVSSSG-D--A---------------------------------------------- 
Chrpic-Dbx1          --SAFSSHSSFLVEDLIRISR---PASYLPR---NGPPP-SMSPPASGTSTARTDTVTS-EL---------------VSCNTSSARRVCSPQTSVSSNND-------------------------------------------------- 
Xenlae-Dbx1          --TALSNHTSFLIEDLIRISR---PAGFLPRAVPPP-SMSPPTSESPNCM----------------------------SETSDLARREGPNQTSISSNNS---S---------------------------------------------- 
Xentro-Dbx1          --TALSSHTSFLIEDLIRISR---PAGFLPRAVPPP-SMSPPTSDSPTSL----------------------------SEVPDLARREAP--TSISSNNS---S---------------------------------------------- 
Latcha-Dbx1          --STFSGQSSFLVEDLLRFSR---PASYLPR---TVPPP-SMSPPTSVTPTALTDSVTT-NH---------------ISTSASSTRGTCSPQTTISSNNDP------------------------------------------------- 
Danrer-Dbx1b         --SAFTTHSSFLVEDLLRISR---PAAFMHR---SIPSPSASPPATGVTTLNTTSSAVHVAM------------------STALAKRSSSPQTSISSDP--------------------------------------------------- 
Leueri-Dbx1          AFSSHAMGASFLVEDLLKISR---PVSYLPR---------SVAPSASDAATAPSPATT--ER---------------HRTASPVTSGICASHSSISPANES------------------------------------------------- 
Petmar-Dbx           HQPSRTLGASFLIDELLRINR-GPPGGY----------GHGRQGPAPPPNASPPAGLLP-------------------PEPLCSAAGACMPVTTASDAA--------------------------------------------------- 
Cioint-Dbx           NFSIYSEYLQMATEQIVHGGV---PSRFGLP---NFPMKYFESPLSVVQNNFDQSEYSSSAN------------RFEDQKNLNGIKRPLSLLTLADKHELSIADEDVANSDTNHTRQPTDATKLRCQYSRNGWNFQESNFRDQVLKTSTE 
Braflo-Dbx           --PATTCSTPFYVDDILRGYHTHAGGCYLP----SLPAMSTASGPTSLRSSPYELSHSN-------------------GCLSTRKFAGVNGTSPHHSGNN-------------------------------------------------- 
Ptyfla-Dbx           ---AST---SFYVENLLRNSA---PNCC---GIARPLPTTQSATTVPT------------------------------LCMNSDCRT-CTSTAVSTTAA-ETTG---------------------------------------------- 
Sackow-Dbx           --ST-----SFYVENLLRNSA---PACCIARPIPTTQTATTVSTL------------CM-----------------------HSDCRTCSSSAAVTTAASETSG---------------------------------------------- 
Strpur-Dbx           --IANSTT-SFLVKDILSSRAPIHKPIPRHPASCTTCNCLRQQKDQLTPNSSSNGNNNNNNNNNHTSQHQDHQHHYHHPHPPHGDRSSSSPPHPPHPPPSVSSSSSASSSHSHNHLPTSSSAPSTQSHHQLPLPLTSSFHPAHHHTPQRL 
Patmin-Dbx           VVQPAGSTTSFLVKDILSHRTPIHKPIPKHPASCTTCNCLRQTQQQQQQ----------------------QQQHHHQRNGSAEGRGDGSPSPAAGAGVVAACEH--------------------------------------------- 
Lotgig-Dbx           --PAVTGSASFLVENLLRERT-----------------SGFLARPQHG-----------------------------QVQNLAQSERDSPPTSAASST---------------------------------------------------- 
Octbim-Dbx           PPAVTTASTSFLVENLLRDRTPPLISRCAAAMTTNSNPASSPSPSAAAAAAAAAAAAAAAAAAATAASSSANTTCTTTTTTTNNNHNNFNNNNINNNNTSLTLVNTPPSLHHPSHTSSHLSSSSSSSPSSSLSSSLSLTSPSSSLSSSSS 
Linana-Dbx           --SGGLSS-SFLVDNLLGAQRTPPQGPY---GVIRSPPVTTVSVHDRIYSIPQLDRGYHMYADKLSCNLSPRPLHQSPASLKTQGKDVQQEEKTQSQVAHGDSARMRHNTDRECSNSRALSNNNNNGSNDRDASSSSSKHVNSANDN--- 
Pladum-Dbx           ---AFVPGTSFLVENLLQRDRAPMPPSSAPLHTPAPPISLPSSGSCPPPPCSSPPPSS--------------------SPPTSPPSSLPSPPPPLPPPSQ---K---------------------------------------------- 
Captel-Dbx           ------GPSPFLVESLLRERQLHAHGLFAPPPPMHVRRALGLNVGSPSPPRKTSDSPSPSPPLLHKERRASCTD---DEEGVKSSMDDATSTRVESPDSE-------------------------------------------------- 
Dromel-Dbx           ------MSTAFLVDSLLHAQQ---TYTESQLKSSLSEMLLSRRSATGTDAADPADQPTESCGCSKLRGYKCDKCREIRAESVESIEEMDEDDGSSSADEIVADIGSVEGEEPHMDLDEELEEELADVDEPVSIKLPKVEPKETSMPTVGN 
Nasvit-Dbx           -------TSPFLMENLLQSKAAPGADLTSLTLNWAASLVARQRERECEANSRHSREKLSPSGASQDQLDQRSSSGARGTDRGNPMIECGVREQQQHQQRGSSGRMVDRQNDRMHMIRDKDGGMERGQIAYVDVDNERIDGHVIVDRLCAM 
Dappul-Dbx           ------------------------------------------------------------------------------------------------------------------------------------------------------ 
Strmar-Dbx           --SNT----SFYVENLLRDRS------SVP-TSPPGYLARPIPVSSATCVQCHHHHHHH--------------------------HHLADAVASATGASK-------------------------------------------------- 
Homsap-Dbx2          ---FGNLGKSFLIENLLRVGG-----------APTPRLQPPAPHDPATALATAGAQLRP-------------------LPASPVPLKLCPAAEQVSPAGA-------------------------------------------------- 
Macfas-Dbx2          ---FGNLGKSFLIENLLRVGG-----------APTPGLQPPAPHGPATALATAGAQLRP-------------------LPASPVPLKLCAAAEQVSPAGA-------------------------------------------------- 
Musmus-Dbx2          ---FGSLGKSFLIENLLRAGA---GPTH---APPPPRPAPGPECPQLRP-----------------------------LPASPVPLKLCPAG---------------------------------------------------------- 
Mondom-Dbx2          ---FGNLGKSFLIENLLRPGD------------DSPQCLTAPSSSNRSIAAVAVHNSEI---------------LEHPLSSSPVPLKLCNGAEQVGS----------------------------------------------------- 
Latcha-Dbx2          ---LGDVGKSFLIENLLKEAP---PFRQ--------------------------------------------------LPANPVPLKLCPSAEQITSTGG-------------------------------------------------- 
Galgal-Dbx2          ---FGSLGKSFLIENLLRAGA-----------PQSPAQLRP-------------------------------------LPASPVPLKLCPAAEQISPSGG-------------------------------------------------- 
Pytbiv-Dbx2          ---FGNLGKSFLIENLLRAGE-------------GPPRLPPR------------------------------------LPATPIPLKLREPMRVAGEG---------------------------------------------------- 
Chrpic-Dbx2          ---SGNLGKSFLIENLLRAAP------------PSP------------------------------------------ARPLPGPLTLCPAAEPASPAGG-------------------------------------------------- 
Xenlae-Dbx2          ---FGNLGRSFLIDDLLKDVD------------PLAMKCLP-------------------------------------VAYSHLPVTQCPTTEQFSPLGS-------------------------------------------------- 
Xentro-Dbx2          ---FGNLGRSFLIDDLLKDVD------------PLAMKCLP-------------------------------------VAYSHVPVTQCPTKEQFSSLGS-------------------------------------------------- 
Danrer-Dbx2          ---FGRSGKSFLMENLLRSTP----------------------------------------------------------------VQIYRTNTGPGSENR-------------------------------------------------- 
Leueri-Dbx2          ---LHNLGKSFLIDNLLRING------------PLNPPLRP-------------------------------------IPANPVPVKVAQTAEQIGPSGG-------------------------------------------------- 

                             310       320       330       340       350       360       370       380       390       400       410       420       430       440       450              
                     ....|....|....|....|....|....|....|....|....|....|....|....|....|....|....|....|....|....|....|....|....|....|....|....|....|....|....|....|....|....|
Homsap-Dbx1          ------------------------------------------------------------------------------------------------------------------------------------------------------ 
Macfas-Dbx1          ------------------------------------------------------------------------------------------------------------------------------------------------------ 
Musmus-Dbx1          ------------------------------------------------------------------------------------------------------------------------------------------------------ 
Mondom-Dbx1          ------------------------------------------------------------------------------------------------------------------------------------------------------ 
Galgal-Dbx1          ------------------------------------------------------------------------------------------------------------------------------------------------------ 
Pytbiv-Dbx1          ------------------------------------------------------------------------------------------------------------------------------------------------------ 
Chrpic-Dbx1          ------------------------------------------------------------------------------------------------------------------------------------------------------ 
Xenlae-Dbx1          ------------------------------------------------------------------------------------------------------------------------------------------------------ 
Xentro-Dbx1          ------------------------------------------------------------------------------------------------------------------------------------------------------ 
Latcha-Dbx1          ------------------------------------------------------------------------------------------------------------------------------------------------------ 
Danrer-Dbx1b         ------------------------------------------------------------------------------------------------------------------------------------------------------ 
Leueri-Dbx1          ------------------------------------------------------------------------------------------------------------------------------------------------------ 
Petmar-Dbx           ------------------------------------------------------------------------------------------------------------------------------------------------------ 
Cioint-Dbx           TIPKQQYE---------------------------------------------------------------------------------------------------------------------------------------------- 
Braflo-Dbx           ------------------------------------------------------------------------------------------------------------------------------------------------------ 
Ptyfla-Dbx           ------------------------------------------------------------------------------------------------------------------------------------------------------ 
Sackow-Dbx           ------------------------------------------------------------------------------------------------------------------------------------------------------ 
Strpur-Dbx           PLHHGSHHRHQIPSPGGSPGANNHHQPPQPALPS-------------------------------------------------------------------------------------------------------------------- 
Patmin-Dbx           ------------------------------------------------------------------------------------------------------------------------------------------------------ 
Lotgig-Dbx           ------------------------------------------------------------------------------------------------------------------------------------------------------ 
Octbim-Dbx           SLSSSSPSSSSLLSSVNPSSNCSSLSTLSLHIPGNNNNNNNNAGCNSSNSNSSSSSNSSSSSSSSNSSNCNNNNSNNNNNSSNNGSSGGVSNGNINGMGVMGIVGVCGGGGGGGSGGGSVGSDSIGSSRDGSGSEGVGGMGCNNSNGAGS 
Linana-Dbx           ------------------------------------------------------------------------------------------------------------------------------------------------------ 
Pladum-Dbx           ------------------------------------------------------------------------------------------------------------------------------------------------------ 
Captel-Dbx           ------------------------------------------------------------------------------------------------------------------------------------------------------ 
Dromel-Dbx           TILKDTNS---------------------------------------------------------------------------------------------------------------------------------------------- 
Nasvit-Dbx           ERLCNERIENRSSSAVDSCNSNVDEDQIGIEMDGGSLQAEREKDDGGILGGERVTALQADRRYDLGCRNVMHTSDEMTIQGEREVAVERVVDRIGERTTTAACSCGDEQCLGPACRTIQEKEK--------------------------- 
Dappul-Dbx           ------------------------------------------------------------------------------------------------------------------------------------------------------ 
Strmar-Dbx           ------------------------------------------------------------------------------------------------------------------------------------------------------ 
Homsap-Dbx2          ------------------------------------------------------------------------------------------------------------------------------------------------------ 
Macfas-Dbx2          ------------------------------------------------------------------------------------------------------------------------------------------------------ 
Musmus-Dbx2          ------------------------------------------------------------------------------------------------------------------------------------------------------ 
Mondom-Dbx2          ------------------------------------------------------------------------------------------------------------------------------------------------------ 
Latcha-Dbx2          ------------------------------------------------------------------------------------------------------------------------------------------------------ 
Galgal-Dbx2          ------------------------------------------------------------------------------------------------------------------------------------------------------ 
Pytbiv-Dbx2          ------------------------------------------------------------------------------------------------------------------------------------------------------ 
Chrpic-Dbx2          ------------------------------------------------------------------------------------------------------------------------------------------------------ 
Xenlae-Dbx2          ------------------------------------------------------------------------------------------------------------------------------------------------------ 
Xentro-Dbx2          ------------------------------------------------------------------------------------------------------------------------------------------------------ 
Danrer-Dbx2          ------------------------------------------------------------------------------------------------------------------------------------------------------ 
Leueri-Dbx2          ------------------------------------------------------------------------------------------------------------------------------------------------------ 
                             460       470       480       490       500       510       520       530       540       550       560       570       580       590       600              
                     ....|....|....|....|....|....|....|....|....|....|....|....|....|....|....|....|....|....|....|....|....|....|....|....|....|....|....|....|....|....|
Homsap-Dbx1          -------------------------------------------------------------------------------------TFLKFGVNAILSSGPRTETSPALL----------------------------------------- 
Macfas-Dbx1          -------------------------------------------------------------------------------------TFLKFGVNAILSSGPRTETSPALL----------------------------------------- 
Musmus-Dbx1          -------------------------------------------------------------------------------------TFLKFGVNAILSSAPRRETSPALL----------------------------------------- 
Mondom-Dbx1          -------------------------------------------------------------------------------------TFLKFGVNAILSSSPRTETSPALL----------------------------------------- 
Galgal-Dbx1          -------------------------------------------------------------------------------------TFLKFGVNAILSSTPRAESSPALL----------------------------------------- 
Pytbiv-Dbx1          -------------------------------------------------------------------------------------TFLKFGVNAILSSPPRTETSPALI----------------------------------------- 
Chrpic-Dbx1          ------------------------------------------------------------------------------------STFLKFGVNAILSSTPRAETSPALL----------------------------------------- 
Xenlae-Dbx1          -------------------------------------------------------------------------------------PFLKFGVNAILSSSPRTESAQVLL----------------------------------------- 
Xentro-Dbx1          -------------------------------------------------------------------------------------TFLKFGVNAILSASPRTETCPALP----------------------------------------- 
Latcha-Dbx1          -------------------------------------------------------------------------------------SFLKFGVNAILSSAPRSDSSSSLL----------------------------------------- 
Danrer-Dbx1b         -------------------------------------------------------------------------------------NYLKFGVNAILASTTR-NASPPPP----------------------------------------- 
Leueri-Dbx1          -------------------------------------------------------------------------------------NYLKFGVHAILSSTPRTESSHPLI----------------------------------------- 
Petmar-Dbx           -------------------------------------------------------------------------------------TALKFGMRAILSSPPKS-----DPSTHFM------------------------------------ 
Cioint-Dbx           ------------------------------------------------------------------------------------KPHLKFGVSTILSLDTISKNQS----------------------SLNSEKTQPPTDCQHGHVQDSH 
Braflo-Dbx           ------------------------------------------------------------------------------------GTYLKFGVNAILSGK---------------------------------QGSPPSP----------- 
Ptyfla-Dbx           -------------------------------------------------------------------------------------TYLKFGVNAILSSATTST----GACTSAF------------------------------------ 
Sackow-Dbx           -------------------------------------------------------------------------------------TYLKFGVNAILSSATTST----GANTSAF------------------------------------ 
Strpur-Dbx           -------------------------------------------------------------------------------------SFLKFGVNSLLSPHSLSPHVSQACTASIGHSAFSDLRPVIA------------------------ 
Patmin-Dbx           ------------------------------------------------------------------------------------GFGLKFGVNAILSAEACS------------------------------------------------ 
Lotgig-Dbx           -------------------------------------------------------------------------------------PFLKFGMNAILGKDSPKSSST--TG-----------SPVIGGSISYTAGSPTLPTYSTS------ 
Octbim-Dbx           FDCEGNVGGGNHHHSHHNNTLNNNNHNSPHNNNNNNNTTSLAGAVSAAAAARSAVAAAEPVTGRTTPSCPTGSVGSMAAGGGVTTPFLKFGMNAILAPDTTPKQENPVFFLLPDGIHTTVIPTRIWQGSAAAINTSVSAFSSCHPSLSSL 
Linana-Dbx           -------------------------------------------------------------------------------------TRLKFGVNAILSLPSKAKEPKTSINGAFSSY----------------HPVPPDFFPLPEHLQHKS 
Pladum-Dbx           -------------------------------------------------------------------------------------PFLKFGVTAILARETSPKNASPGGGSAFHAYKTQSTGAGRKSPGSPGRRSPASPGAPDDRQKHLT 
Captel-Dbx           ------------------------------------------------------------------------------------KPFLKFGVNAILAPNTRVTSPK-QHN-----------SHGCRLSSSPSSSSPSPRSSLPSPS---- 
Dromel-Dbx           ------------------------------------------------------------------------------------KPILKFSVSAILGDTREGVRVRNEFMQPQHIWPYLQQNFMQQHSHQYQQHHQQQQPHSHPQHQPLA 
Nasvit-Dbx           -------------------------------------------------------------------------------------PQLKFSVNAILGGNHDRRPHS------------------ENFQGLPPEAIPAFLQNLQNSASYNI 
Dappul-Dbx           ------------------------------------------------------------------------------------KPYLKFGVSAILGLDIQPRTPSPIDNGSSPLLRPEESPQPLDMKTELFHHHHIPHLHHHHSSSLVA 
Strmar-Dbx           -------------------------------------------------------------------------------------PYLKFGVSAILDGQDMV------------------------------SSSP-------------- 
Homsap-Dbx2          -------------------------------------------------------------------------------------PYGTRWAFQVLSPSADSARLP-GRAPGDRDCTFQP------------------------------ 
Macfas-Dbx2          -------------------------------------------------------------------------------------PYGTRWAFQVLSPSMDSARLP-GRAPGDRDCTFQP------------------------------ 
Musmus-Dbx2          -------------------------------------------------------------------------------------PFGVRWAFQMPP----------GRAPGERDSAFQP------------------------------ 
Mondom-Dbx2          -------------------------------------------------------------------------------------PYRTHWAFQVFNPST-------DSRPLPGQGDQGGAFQL-------------------------- 
Latcha-Dbx2          -------------------------------------------------------------------------------------PYSTRWAFQVLNPTASQ-----PAHSANRAIFYHTP----------------------------- 
Galgal-Dbx2          -------------------------------------------------------------------------------------PYPTRWAFQVLNPSAAD-----GGRLPARAPAADRGGVFPP------------------------ 
Pytbiv-Dbx2          ------------------------------------------------------------------------------------AFPGRAWPFQALNASAAD-----GGAPADRGGAFPA------------------------------ 
Chrpic-Dbx2          -------------------------------------------------------------------------------------PGPARWACPRLHPSAAASSGLPARAPDRATGIFPP------------------------------ 
Xenlae-Dbx2          -------------------------------------------------------------------------------------TYPRAWTFQLLRTSDRWQ----AFYPQPFTGNV-------------------------------- 
Xentro-Dbx2          -------------------------------------------------------------------------------------TYPRTWTFQLLRTSDRRQ----PFYPHPFTGDV-------------------------------- 
Danrer-Dbx2          ------------------------------------------------------------------------------------QHTQRIHTPAVIMSTV-------------------------------------------------- 
Leueri-Dbx2          -------------------------------------------------------------------------------------PHPTRSSFQLVNPS--------DNQPAPVTSTSQTVTY--------------------------- 

                             610       620       630       640       650       660       670       680       690       700       710       720       730       740       750              
                     ....|....|....|....|....|....|....|....|....|....|....|....|....|....|....|....|....|....|....|....|....|....|....|....|....|....|....|....|....|....|
Homsap-Dbx1          ------------------------------------------------------------------------------------------------------------------------------------------------------ 
Macfas-Dbx1          ------------------------------------------------------------------------------------------------------------------------------------------------------ 
Musmus-Dbx1          ------------------------------------------------------------------------------------------------------------------------------------------------------ 
Mondom-Dbx1          ------------------------------------------------------------------------------------------------------------------------------------------------------ 
Galgal-Dbx1          ------------------------------------------------------------------------------------------------------------------------------------------------------ 
Pytbiv-Dbx1          ------------------------------------------------------------------------------------------------------------------------------------------------------ 
Chrpic-Dbx1          ------------------------------------------------------------------------------------------------------------------------------------------------------ 
Xenlae-Dbx1          ------------------------------------------------------------------------------------------------------------------------------------------------------ 
Xentro-Dbx1          ------------------------------------------------------------------------------------------------------------------------------------------------------ 
Latcha-Dbx1          ------------------------------------------------------------------------------------------------------------------------------------------------------ 
Danrer-Dbx1b         ------------------------------------------------------------------------------------------------------------------------------------------------------ 
Leueri-Dbx1          ------------------------------------------------------------------------------------------------------------------------------------------------------ 
Petmar-Dbx           ------------------------------------------------------------------------------------------------------------------------------------------------------ 
Cioint-Dbx           ---------TQD------------------------------------------------LRSTLITQACHPYSQHP-------------------------------------------------VTQYPHYVTHGTY----------- 
Braflo-Dbx           ---------SVE------------------------------------------------SCRCAFYPQPRSY--PA-------------------------------------------------AETYLSPVNKVPYTESPTTF---- 
Ptyfla-Dbx           ------------------------------------------------------------------------------------------------------------------------------------------------------ 
Sackow-Dbx           ------------------------------------------------------------------------------------------------------------------------------------------------------ 
Strpur-Dbx           ------------------------------------------------------------------------------------------------------------------------------------------------------ 
Patmin-Dbx           ------------------------------------------------------------------------------------------------------------------------------------------------------ 
Lotgig-Dbx           ---------CGG------------------------------------------------KACGGYTLHGLPC-NSCNTTRQPTL----------------------------------------------------------------- 
Octbim-Dbx           TSVACSKSSCTLGNQGITCAGCNPRGATLYESHLQG------IVRHPY------F---SATPLL---PIPNT------------------------------------------------------------------------------ 
Linana-Dbx           PYGGYPVSLPYT------------------------------------------------PYAGHIHYAPHPY----------------------------------------------------------------------------- 
Pladum-Dbx           EPQIPHSRNYEPNTSATTMADFKQYTYERQNMHTSVPTSHMTSDFDTRTVSTHSSQIKTLMAPHPMMQGFPPRIFSCPHSKLGPCLS--------------------------------------------------------------- 
Captel-Dbx           ---------GRP------------------------------------------------ALSSAFGPYPFPLHASSLQQAAAAMAASKGGCHPMPPCNGCAPTPPRGGDVMSGFMRHPY-------------------------F---- 
Dromel-Dbx           VPGHQHSHPHSHHHHHHHPGAGHAHFPHPAFLTHQLPPHQHHQQQQHVQQQHPGNSCQPQTASSSSSPGSTISDSDNNHGASTANGNGSGTGNSGQDARPQDARPHELANSNPDEDSSASRRLTQDKQQQQQLLGAGGTGGGGGGGHGHG 
Nasvit-Dbx           AK-------PIA------------------------------------------------RPAAYHPY-HRPSHQPPQRHLPPNA----------------------------------------------------------------- 
Dappul-Dbx           DHGDP-------------------------------------------------------KTPNVTTFHPVYH-----------------------------------------------------LHSYFHPLIQQQQ----------P 
Strmar-Dbx           ------------------------------------------------------------------------------------------------------------------------------------------------------ 
Homsap-Dbx2          ------------------------------------------------------------------------------------------------------------------------------------------------------ 
Macfas-Dbx2          ------------------------------------------------------------------------------------------------------------------------------------------------------ 
Musmus-Dbx2          ------------------------------------------------------------------------------------------------------------------------------------------------------ 
Mondom-Dbx2          ------------------------------------------------------------------------------------------------------------------------------------------------------ 
Latcha-Dbx2          ------------------------------------------------------------------------------------------------------------------------------------------------------ 
Galgal-Dbx2          ------------------------------------------------------------------------------------------------------------------------------------------------------ 
Pytbiv-Dbx2          ------------------------------------------------------------------------------------------------------------------------------------------------------ 
Chrpic-Dbx2          ------------------------------------------------------------------------------------------------------------------------------------------------------ 
Xenlae-Dbx2          ------------------------------------------------------------------------------------------------------------------------------------------------------ 
Xentro-Dbx2          ------------------------------------------------------------------------------------------------------------------------------------------------------ 
Danrer-Dbx2          ------------------------------------------------------------------------------------------------------------------------------------------------------ 
Leueri-Dbx2          ------------------------------------------------------------------------------------------------------------------------------------------------------ 

                             760       770       780       790       800       810       820       830       840       850       860       870       880       890       900              
                     ....|....|....|....|....|....|....|....|....|....|....|....|....|....|....|....|....|....|....|....|....|....|....|....|....|....|....|....|....|....|
Homsap-Dbx1          ---------QS-VPPKTF---AFPY----FEGSFQPFIRSSY-----FPASSSVVPIPGTFSWPLAARGKPRRGMLRRAVFSDVQRKALEKMFQKQKYISKPDRKKLAAKLGLKDSQVKIWFQNRRMKWRNSKERELLSSGGCREQTLPT 
Macfas-Dbx1          ---------QS-VPPKTF---AFPY----FEGSFQPFIRSSY-----FPASSSVVPIPGTFSWPLAARGKPRRGMLRRAVFSDVQRKALEKMFQKQKYISKPDRKKLAAKLGLKDSQVKIWFQNRRMKWRNSKERELLSSGGCREQTLPT 
Musmus-Dbx1          ---------QS-PPPKTF---AFPY----FEGSFQPFIRSSY-----FPASSSVVPIPGTFSWPLAARGKPRRGMLRRAVFSDVQRKALEKTFQKQKYISKPDRKKLASKLGLKDSQVKIWFQNRRMKWRNSKERELLSSGGCREQTLPT 
Mondom-Dbx1          ---------QS-VPPKSF---SFPY----FEGSFQPFIRSSY-----FPASSSVVPIPGTFSWPLAARGKPRRGMLRRAVFSDVQRKALEKMFQKQKYISKPDRKKLAAKLGLKDSQVKIWFQNRRMKWRNSKERELLSNGGCREQTLPT 
Galgal-Dbx1          ---------QS-VPPKTF---SFPY----FEGSFQPFIRSSY-----FPAASAVVPIPGTFSWPLAARGKPRRGMLRRAVFSDVQRKALEKMFQKQKYISKPDRKKLAAKLGLKDSQVKIWFQNRRMKWRNSKERELLSSGGCREQTLPT 
Pytbiv-Dbx1          ---------QN-VPVKTF---SFPY----FEGSFQPFIRSSY-----FPA-SSVVPIPGTFSWPLAARGKPRRGMLRRAVFSDVQRKALEKMFQKQKYISKPDRKKLAAKLGLKDSQVKIWFQNRRMKWRNSKERELLSSGGCREQTLPT 
Chrpic-Dbx1          ---------PS-VPPKTF---SFPY----FEGSFQPFIRSSY-----FPASSSVVPIPGTFSWPLAARGKPRRGMLRRAVFSDVQRKALEKMFQKQKYISKPDRKKLAAKLGLKDSQVKIWFQNRRMKWRNSKERELLSSGGCREQTLPT 
Xenlae-Dbx1          ---------PS-AHPKPF---TFPY----FEGSFQPFIRSSY-----FPASSSVVPIPGTFSWPLVARGKPRRGMLRRAVFSDVQRKALEKMFQKQKYISKPDRKKLAGKLGLKDSQVKIWFQNRRMKWRNSKERELLSSGGCREQTLPT 
Xentro-Dbx1          ---------PSVAPPKAF---AFPY----FEGSFQPFIRSSY-----FPASSSVVPIPGTFSWPLAARGKPRRGMLRRAVFSDVQRKALEKMFQKQKYISKPDRKKLAGKLGLKDSQVKIWFQNRRMKWRNSKERELLSSGGCREQTLPT 
Latcha-Dbx1          ---------QT-VGPKSF---TFPY----FEGSFQPFIRSSY-----FPASSSVVPIPGTFSWPLAARGKPRRGMLRRAVFSDVQRKALEKMFQKQKYISKPDRKKLAAKLGLKDSQVKIWFQNRRMKWRNSKERELLSSGGCREQTLPT 
Danrer-Dbx1b         ---------VQGMNAKTF---PFPC----FDGSFHPFIRASY-----FPASSSAVPIPGTFAWPLTARGKPRRGMLRRAVFSDVQRKALEKMFQKQKYISKPDRKKLATKLGLKDSQVKIWFQNRRMKWRNSKERELLSSGGCREQTLPT 
Leueri-Dbx1          ---------QG-ISPKAF----FPY----FEGSFQPFIRSTY-----FPASSSVVPIPGTFSWPLAARGKPRRGMLRRAVFSDVQRKALEKMFQKQKYISKPDRRKLAAKLGLKDSQVKIWFQNRRMKWRNSKERELLSAGGCREQTLPT 
Petmar-Dbx           ---------QE-YSTKAL---PYSY----FDGHFQQFIRSAY-----YPA-SAVIPMPGSFSWALAARGKPRRGMLRRAVFSDVQRKALEKMFQKQKYISKPDRRKLATKLGLKDSQVKIWFQNRRMKWRNSKERELLSAGGCREQTLPT 
Cioint-Dbx           ------------------------------------------------VHSNPVAPILPT-WYQGAVRGRGRRGMLRRAVFSDNQRKALEKKFQLQKYIGKPDRKKLALKLGLKDSQVKIWFQNRRMKWRNSQERQMISSSLSTENN--- 
Braflo-Dbx           ----------------------------------------------------PIFNACHWGAPPGLHRGKPRRGMLRRAVFSDFQRKSLEKMFQKQKYISKQDRRKLAEKLGLKDSQVKIWFQNRRMKWRNSKERELLSTGGSRESTIPN 
Ptyfla-Dbx           ---------ST-YAPKPVPSIPRPF----YDSPLQPLTRGPY-----FPA-STVIPVPGTYAWPATARGKPRRGMLRRAVFSDAQRKGLEKKFQQQKYISKPDRRKLAAKLGLKDSQVKIWFQNRRMKWRNSKERELLSAGGSRESTLPN 
Sackow-Dbx           ---------STYSAGKPLVSLPRPF----YDSPVQPLSRNPY-----FPA-STVIPVPGTYAWPATARGKPRRGMLRRAVFSDAQRKGLEKKFQQQKYISKPDRRKLASKLGLKDSQVKIWFQNRRMKWRNSKERELLSSGGSRESTLPN 
Strpur-Dbx           ---------SLGTLHRSFYENS-------FHQSPTSAPRNPF----LQVPPSSVIPVPGTFPWPGAARGKPRRGMLRRAVFSDAQRKGLEKRFQQQKYISKPDRKKLAAKLGLKDSQVKIWFQNRRMKWRNSKERELLSAGGSRESTLPN 
Patmin-Dbx           ---------SQGDVHNLIWTDASSL----MQTQSQLFQTCLHFCFVFPVTPSSVIPVPGTFPWPGAARGKPRRGMLRRAVFSDAQRKGLEKKFQQQKYISKPDRKKLAAKLGLKDSQVKIWFQNRRMKWRNSKERELLSSGGSRESTLPN 
Lotgig-Dbx           -----------------------------YDTHFTNMLRPPYL-----SGSF--LPMPNAFTFLSNMRGKPRRGMLRRAVFSDAQRKGLEKMFQKQKYISKPDRKKLASKLGLKDSQVKIWFQNRRMKWRNSKERELLSSGGSRDSTLPN 
Octbim-Dbx           ------------------------------------------------------------FSFLNNIRGKPRRGMLRRAVFSDLQRKGLEKMFQKQKYISKPDRKKLAAKLGLKDSQVKIWFQNRRMKWRNSKERELLSSGGTRESTLPN 
Linana-Dbx           -----------------------------PHPSPPIFLKNPYL-----SGGM--LPLPNALSILQGMRGKPRRGMLRRAVFSDAQRKGLEKMFQKQKYISKPDRKKLAAKLGLKDSQVKIWFQNRRMKWRNSKERELMSAGGTREATIPT 
Pladum-Dbx           -----------------------------CRHMMYEPQNLPAFVKTPFNYSPSLFPMPNALSLLSSMRGKPRRGMLRRAVFSDAQRKGLEKMFQKQKYISKPDRKKLADKLGLKDSQVKIWFQNRRMKWRNSKERELLSSGGSRDQTLPN 
Captel-Dbx           ----------------------------------------------PGSALFPGIPNAAAFSFLNGMRGKPRRGMLRRAVFSDFQRKGLEKMFQKQKYISKPDRKKLAEKLGLKDSQVKIWFQNRRMKWRNSKERELLSSGGSREATIPS 
Dromel-Dbx           HPTPPPPPPPPVIAKPMPSRPTPFLPHTLNHPHLHSLLAHCRNPYMSVGAQVFPLPPGQGFPWAHSTRGKPRRGMMRRAVFSDSQRKGLEKRFQQQKYISKPDRKKLAERLGLKDSQVKIWFQNRRMKWRNSKERELLASGGSRDQTLPN 
Nasvit-Dbx           --------HHTLQQLFYRGPYLTVAGSGTGGHHPGAPGGGTAFPGAIQGGLGDFASTGLVFPWATNARGKPRRGMMRRAVFSDLQRRGLEKRFQIQKYISKPDRKKLAEKLGLKDSQVKIWFQNRRMKWRNSKERELLATGGSREQTLPN 
Dappul-Dbx           ---------------------------------------------------------------AHSANKPNYPGMMRRAVFSELQRRGLERRFQIQKYISKPERKKLAEKLGLKDSQVKIWFQNRRMKWRNSKERELLANGGSRSQTLPT 
Strmar-Dbx           ------------------------------------LVNGSS---------------------TTAVRGKPRRVMLRRAVFSDVQRRELEKRFHIQKYISKPDRKKLAEKLGLKDSQVKIWFQNRRMKWRNSKERELLASGGSRDQTLPN 
Homsap-Dbx2          ---------SAPAPSKPFLLSTPPFYSACCGGSCRRPASSTA------FPREESM----LPLLTQDSNSKARRGILRRAVFSEDQRKALEKMFQKQKYISKTDRKKLAINLGLKESQVKIWFQNRRMKWRNSKEKEVLSNRCIQEVGLQE 
Macfas-Dbx2          ---------SAPAPSKHFLLSAPPFYSACCGGSCRRTASSAA------FPREESV----LPLLTQDSNSKARRGILRRAVFSEDQRKALEKMFQKQKYISKTDRKKLAINLGLKESQVKIWFQNRRMKWRNSKEKEVLSNRCIQEVGLQE 
Musmus-Dbx2          ---------SAPVPSKPFLLSAPPFYSACCGGSCRRPASPTA------FSREEHG----LPLLTQDSNSKARRGILRRAVFSEEQRKALEKMFQKQKYISKTDRRKLAVSLGLKESQVKIWFQNRRMKWRNSKEKEVLSSRCLQEVSLQE 
Mondom-Dbx2          ---------AVPASSKLFFLRAPQFYSTCCGGSCQHPASPTA------LPREDSM----LPLLTEDSNSKAQRGILRRAVFSDDQRKSLEKMFQKQKYINKTDRKKLSISLGLKESQVKIWFQNRRMKWRNCKEKEALSNQYAKEEGIQE 
Latcha-Dbx2          ---------AATATSKHLFPRVPPYSFMCCGGSCQHLASPTA------FPKAESV----LPLWTQDINFRTRRGILRRAVFSEEQRKELEKMFLKQKYISKTDRKKLAVTLELKESQVKIWFQNRRMKWRNSKEKEMLANGCSHEEILQE 
Galgal-Dbx2          ---------AATALSKHFFLRAPPFYSACCGGSCQHPASPTA------FPREESV----LPLLTQESNSKARRGILRRAVFSEDQRKALEKMFQKQKYISKTDRKKLAINLGLKESQVKIWFQNRRMKWRNSKEKEVLSNRCLQE-GLQE 
Pytbiv-Dbx2          ---------AAPALPKHFFLSASPVYLACCGGSCQHPASPMA------FPRQESA----LPLL--THDPRSRRGILRRAVFSEEQRKSLEKMFQKQKYISKTDRKKLALNLGLKESQVKIWFQNRRMKWRNSKEKEVLSNRCLPEEGLQE 
Chrpic-Dbx2          ---------AATVLSKHFFLRAPPFYSACCGGSCQHPVSPTA------FPREESV----LPLLTQDSNSKARRGILRRAVFSEDQRRALEKMFQKQKYISKTDRKKLAINLGLKESQVKIWFQNRRMKWRNSKEKEVLSNRCLQEEGLQE 
Xenlae-Dbx2          ---------YETDFSKYFYLQSVPFYSACCGGSCQHPASPTA------FPRNEML----LPLSGQDANSKTRRVILRRAVFSEEQRKALEKMFQKQKYISKVDRKKLAVKLALKESQVKIWFQNRRMKWRNSKEKSVLLNTFLKEDSAEQ 
Xentro-Dbx2          ---------YETAFSKYCYLQSAPFYSACCGGSCQHPASPTA------FPRNEML----PPLCGQDASSKTRRVILRRAVFSEEQRKSLEKMFQKQKYISKVDRKNLAVNLALKESQVKIWFQNRRMKWRNSKEKSVLSNKFLKEDSAEQ 
Danrer-Dbx2          ---------------------NLPC----CSGSGPVMTTSPL-----------FTKAVGSVLWTPAINSRSRPGILRRAVFSEEQRRELEKTFSKQKYISKTERNRLASELSLKETQVKIWFQNRRMKWRNSREKEISNTHTHMEKLMDW 
Leueri-Dbx2          ---------PDIALSKCF----LPS----FGGSCQQQVSPTD------FPISSSFRNCEFPLWPQVSHNKVRRGILRRVVFSEEQRKSLEKTFQKQKYISKTDRKKLATHLGLKDSQVKIWFQNRRMKWRNTKERELLTQGYSLEHTLQE 
                             910       920       930       940       950       960       970       980       990       1000      1010      1020      1030      1040      1050             
                     ....|....|....|....|....|....|....|....|....|....|....|....|....|....|....|....|....|....|....|....|....|....|....|....|....|....|....|....|....|....|
Homsap-Dbx1          KLNPHPDLSDV------------------------------------------------------------------------------------------------------------------------------GQKGPGNEEEEEG 
Macfas-Dbx1          KLNPHPDLSDV------------------------------------------------------------------------------------------------------------------------------GQKGPGDEEEEEE 
Musmus-Dbx1          KLNPHPDLSDV------------------------------------------------------------------------------------------------------------------------------GQKGPGDEEE--- 
Mondom-Dbx1          KLNPHPDLSDV------------------------------------------------------------------------------------------------------------------------------GKKCPGDEDEEED 
Galgal-Dbx1          KFNPHPDLSDV------------------------------------------------------------------------------------------------------------------------------GKKCSGEEEEEDE 
Pytbiv-Dbx1          KFNPHPDLSDV------------------------------------------------------------------------------------------------------------------------------GKKGTGEEEEEAV 
Chrpic-Dbx1          KFNPHPDLSDV------------------------------------------------------------------------------------------------------------------------------GKKCSEEEEEEEE 
Xenlae-Dbx1          KFNPHPDLSDV------------------------------------------------------------------------------------------------------------------------------SKKSSGEGEEEPL 
Xentro-Dbx1          KFNPHPDLSDV------------------------------------------------------------------------------------------------------------------------------GKKSSGEGEEEPM 
Latcha-Dbx1          KFNPHPDLSDV------------------------------------------------------------------------------------------------------------------------------GKKSAEAQE---- 
Danrer-Dbx1b         KMNPNPDLSDV------------------------------------------------------------------------------------------------------------------------------GKRFEHEAVLR-- 
Leueri-Dbx1          KLNPNPDLSDV------------------------------------------------------------------------------------------------------------------------------GKSSPCSLHSGDE 
Petmar-Dbx           KGNPHPDLSDV-----------------------------------------------------------------------------------------------------------------------------QGRRGALSPGGTTT 
Cioint-Dbx           ------------------------------------------------------------------------------------------------------------------------------------------------------ 
Braflo-Dbx           KANPNPDLSDV------------------------------------------------------------------------------------------------------------------------------GQT---------- 
Ptyfla-Dbx           KSNPNPDLSDV-----------------------------------------------------------------------------------------------------------------------------ADDKKKDLSDSDKE 
Sackow-Dbx           KSNPNPDLSDV-----------------------------------------------------------------------------------------------------------------------------ADDRKHELSDSEKE 
Strpur-Dbx           RSNPNPDLSDV-----------------------------------------------------------------------------------------------------------------------GGACNALLMGNNPDCESDSE 
Patmin-Dbx           KSNPNPDLSDV-----------------------------------------------------------------------------------------------------------------------------AEEKSSPETQHPHD 
Lotgig-Dbx           KNNPNPDLSDV-----------------------------------------------------------------------------------------------------------------------------RDDPSVMINDVDHM 
Octbim-Dbx           KNNPNPDLSDV------------------------------------------------------------------------------------------------------------------------------GKGDESDDIE--- 
Linana-Dbx           RNNPNPDLSDV-----------------------------------------------------------------------------------------------------------------------------SDKLQDKSLPSPTS 
Pladum-Dbx           KDNMNSEFDKE-----------------------------------------------------------------------------------------------------------------------------SGENLNFEEILADI 
Captel-Dbx           KDNPKPDLSDE-----------------------------------------------------------------------------------------------------------------------------LGLHTTSPIRTE-- 
Dromel-Dbx           KNNPNPDLSDAKCDRPLTPLSPSLLSPNGSATPPPPGAVAKDEPPTAAVTPKSPQSVSSRSSPSVGYGLQISSPPPLLTSLKMGDQSASATPTPLPGTTVSSAAASPPGVTASEFQAKINAEMQKQLVAADLKFKLENSIQEAKQRRFEQ 
Nasvit-Dbx           KNNPNPDLSDA-----------------------------------------------------------------------------------------------------------------------------DGDRPRLDLS---- 
Dappul-Dbx           KNNPHPDLSDS-----------------------------------------------------------------------------------------------------------------------------EIERAAH------- 
Strmar-Dbx           KNNPNPDLSDP-----------------------------------------------------------------------------------------------------------------------------EDRKTCGDIECRIG 
Homsap-Dbx2          DPLSRSAL---------------------------------------------------------------------------------------------------------------------------------GFPSPCPSIWDVP 
Macfas-Dbx2          DPLSRSAL---------------------------------------------------------------------------------------------------------------------------------GFPSPCPSIWDVP 
Musmus-Dbx2          DRLARPAV---------------------------------------------------------------------------------------------------------------------------------GCPPQCPSIWEVS 
Mondom-Dbx2          NQISRSTL---------------------------------------------------------------------------------------------------------------------------------GFTSPCSKIWKVS 
Latcha-Dbx2          NTMANSTLNDH-----------------------------------------------------------------------------------------------------------------------------SSHTPIIDTSSTRN 
Galgal-Dbx2          NYLSQSAM---------------------------------------------------------------------------------------------------------------------------------NFASPCPSVWEVS 
Pytbiv-Dbx2          TYVSRCLN---------------------------------------------------------------------------------------------------------------------------------FSSSPCP-VWEMS 
Chrpic-Dbx2          NYLTRSTL---------------------------------------------------------------------------------------------------------------------------------NFTSPCPSIWEVS 
Xenlae-Dbx2          NLSRTVQ----------------------------------------------------------------------------------------------------------------------------------CIHTPC------- 
Xentro-Dbx2          KLSRTVQ----------------------------------------------------------------------------------------------------------------------------------CIHTPC------- 
Danrer-Dbx2          SQSEPAD----------------------------------------------------------------------------------------------------------------------------------DTHTPAQSKHTHT 
Leueri-Dbx2          KHLSTTDSNNG-----------------------------------------------------------------------------------------------------------------------------IANDPDRKSTHLDQ 

                             1060      1070      1080      1090      1100      1110      1120      1130      1140       
                     ....|....|....|....|....|....|....|....|....|....|....|....|....|....|....|....|....|....|.
Homsap-Dbx1          P----------GSPSHRLAYHASS------------DPQHLRDPRLPGPLPPSPAHSSSPGKPSDFSDSE--EEEEG--EEQEEITVS--- 
Macfas-Dbx1          GP---------GSPRHRLAYHASP------------DPPHLRDPRLPEPLPPSPAHSSSPGKPSDFSDSE----EEEEGEEEEEITVS--- 
Musmus-Dbx1          -----------DNPGARLAYHAPA------------DPRH----LLEGPLPASPAHSSSPGKPSDFSDSD--EDEEG--EEDEEITVS--- 
Mondom-Dbx1          EEDENSALGS-ASPRHSLAFHRSP------------EHLHLRD-RLAAQLPPSPSHSSSPSKPSDFSDSE--EEEEEGEGEEEEITVS--- 
Galgal-Dbx1          GP-----MACPPSPRHPLPYHPAP------------Q--HLRD-RLGPQTPPSPSHSSSPSKPSDCSDSE--EEDEEG-EEEEEITVS--- 
Pytbiv-Dbx1          SP------LYPASPRHSLAYHQST------------DHTHQRD-RLDSQMLPSPTHSSSPSKPSDFSDSEEEEEEEGEEEEDEEITVS--- 
Chrpic-Dbx1          VS-----PLCPASPRHTLTYHQSP------------EHLHLRD-RLDSQMPPSPSHSSSPSKPSDFSDSE--EEDDEGEEEEEEITVS--- 
Xenlae-Dbx1          CPG--------NSPAHALPYQCPE-----------------HHLRLDTQLPSSPFNSSSASKPSDFSDSE-----EEGGEQEEEITVS--- 
Xentro-Dbx1          C----------NSPAHSSPYQCPE-----------------HSLRLDAQLPPSPFNSSIASKPSDFSDSE-----EEEGEQEEEITVS--- 
Latcha-Dbx1          -----------EALSPPLSLYTCP------------EHGVR---ELAEAHLPSPCHSCKHS---DFSESE-----------EEEITVS--- 
Danrer-Dbx1b         -----------ESPRAPFCQSRGD-------------------HEFNADLHFKSPSISSKH--SDFSE-----------SEDEEITVS--- 
Leueri-Dbx1          EELGCR-----DSPKSPLCQSPDY-------------------KRIDTHLRLISHQLAADQ-HTYFSEEE---------DGEEEITVS--- 
Petmar-Dbx           AATAVDEIER-QSPSTPLSSSSSS-------------------GCSSGSFRYEEFHEERHL-TASFEDS----------EDDVEISVL--- 
Cioint-Dbx           ---------------------------------------------------------------KVQVLPIPKCVREQTEGATSQVAVDE-- 
Braflo-Dbx           -----------TGASSPLIGSPSP-------------------------------------GVTDQKTPPSSPESPRNEEPAESENKK--- 
Ptyfla-Dbx           L----------DSPTSSPCASPLP-----------------------DDSSDAMHIANGAE------------------SDGEEIKVL--- 
Sackow-Dbx           I----------DSIASSPCASPTA------------------EDHNGDIIVPHDHHIDDMA-DDMIVDDD--DD-----DDEEEIKVL--- 
Strpur-Dbx           KELM-------DTPTSSPCGSPTH--LQHHVRSTHAQEELLMPTMHGHYLHHNEYGVDDDRKIPEFAEVG---------DSDNEIDID--- 
Patmin-Dbx           HH---------HHHHHQHEHQHHGLGQADQGSDLDGEILDSPASSPCSTGETADDDGRRSPAAAGRDGEY--EDDEEDEEEERDIDVM--- 
Lotgig-Dbx           -----------ESNQSQFSPQYSP----------------------EQSSPVCLDHLDRME--ELQQSEE---------DSDEEIDVS--- 
Octbim-Dbx           -----------GSPKNDNSKDLDS--------ISRELSPSAHSPTSSFQEEHNL-DINISESD----------------SEDEEINVS--- 
Linana-Dbx           EE---------QITDRTLAVNCLS------------SSGETQKYPLPGSASLVTSTNDVDSDELLNVTSDSDDDEDEDDVDCEEMEYGPAT 
Pladum-Dbx           SNEDITDEHSVHSLEDSEIHNDIR-------------NDIRNDIRNDIHMRINPES-----------------------DDEEEIDVSH-- 
Captel-Dbx           -----------SNLINSEMYEPLD---------------------LRGSSTHPALIGDMESSKMESEDDE-EDDDEESDVDGEEICVS--- 
Dromel-Dbx           LQQQLHHSPHFSAMREVELAAAAA-------AAQLHHHHQQQQQQQQQQHAQQQHAQQQHQGGSEFMKLYYDDYDDSNSDSDEEISVT--- 
Nasvit-Dbx           --------------------------------------------------------------DVSPLTSPQRPEEQTENEEDEEINVT--- 
Dappul-Dbx           -----------HTSSSSFHHLKST-------------------QQQQQQPTPANESISAT-------------------CCADEIR----- 
Strmar-Dbx           EMDM-------SSCTSPASITSSG-----------DILRGGQILETRRDSQGED-------------------------SDDEEIKVL--- 
Homsap-Dbx2          -----------QQHSSPRWRENSP----------------------EPSERLIQESSGAPPPEANSLQG-ALYLCSEEEAGSKGVLTGAV- 
Macfas-Dbx2          -----------QQHSSPRWRKNSP----------------------EPSERLIQESSGAPPPEANSLQG-ALYLCSEEEAGSKGVLTGAI- 
Musmus-Dbx2          -----------QPHSSPSWREETP----------------------ESAERLTQENSGVL--EADSLRG-TLYLCPEKGPRDKHGLQSTI- 
Mondom-Dbx2          -----------QQHSSSRWRENSP----------------------EPSERLNHENSLQLLPQVNSHQG-PLYLYSEADTEDKAVTAAI-- 
Latcha-Dbx2          -----------TSISVLKCRRSSP---------------------ASIQLISETEKTALIQKVESREKGLCLY--REEYNKRMEAALPL-- 
Galgal-Dbx2          -----------QEQTSPRWKKSPG--------------------------NSERLTSTQPPPRANSSQS-PLYLYPDHDTANKAVTSSD-- 
Pytbiv-Dbx2          -----------QQQSSSRW-QNFP----------------------ECAGRLSNRISLRPPIKPNLSSD-TSYLYRERSNTEKATPS---- 
Chrpic-Dbx2          -----------QQQASPRWRENSA----------------------EPSERLNNRNSAQPPQEADSFQG-TLYLYPEPDTGEKAVTLSV-- 
Xenlae-Dbx2          ------------------------------------------------------------------------------------------- 
Xentro-Dbx2          ------------------------------------------------------------------------------------------- 
Danrer-Dbx2          HTHR--------------------------------------------------------------------------------------- 
Leueri-Dbx2          SPTCKYTALTSMSQSSQSGENRVP----------------------DVKVEILKTSPHSHSIL-----------------HDKYI------ 
