# Supplementary figures and images for: Neuronal fate specification by the Dbx1 transcription factor is linked to the evolutionary acquisition of a novel functional domain
Source: EvoDevo. 2016 Aug 12;7:18. doi: 10.1186/s13227-016-0055-5 (PMC4983035; doi:10.1186/s13227-016-0055-5)

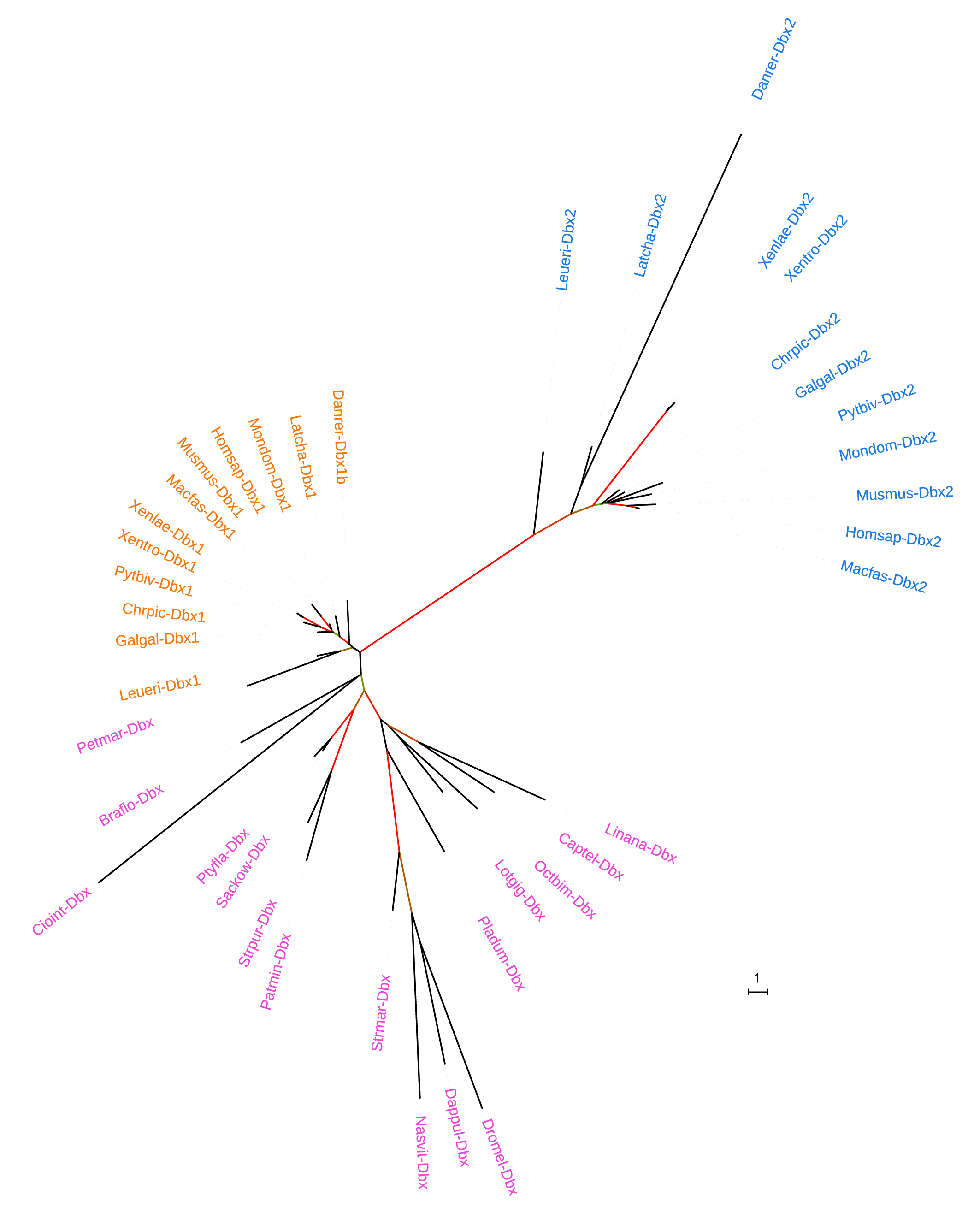

Supplement: Supplementary file 2 — 10.1186/s13227-016-0055-5 Maximum likelihood phylogenetic tree of the Dbx family proteins. Dbx, Dbx1 and Dbx2 sequences are indicated in pink, orange and blue, respectively. aLRT statistical support is color coded (from 70 % in green to 100 % in red). A schematic representation of the functional domains found in each sequence is also reported. The scale bar indicates the branch length that corresponds to the number of substitutions per residue. [file 13227_2016_55_MOESM2_ESM.tif]

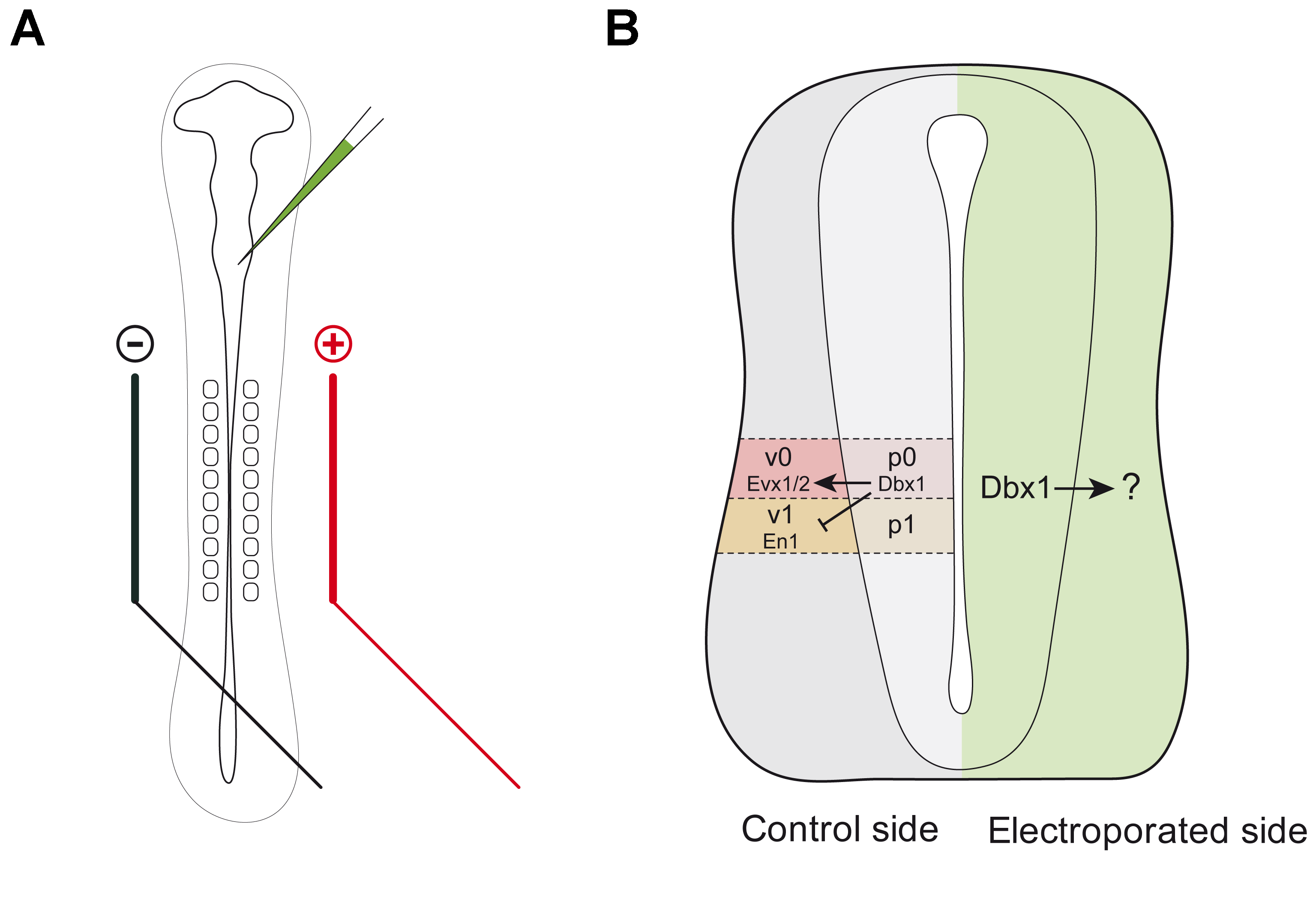

Supplement: Supplementary file 3 — 10.1186/s13227-016-0055-5 Diagram of the electroporation experiments. (A) DNA is injected in the neural tube of developing chick embryos and transfected through electroporation in only one half of the spinal cord. (B) On the control side of the spinal cord, Dbx1 is expressed by p0 progenitors; it favors their differentiation in v0 (Evx1/2+) interneurons and prevents v1 (En1+) fate. On the electroporated side, the fate specification properties of various Dbx1 constructs are assessed by counting the number of v0 and v1 neurons generated relative to the control side. [file 13227_2016_55_MOESM3_ESM.tif]
